# Supplementary material for: Culture Dimensionality Modulates Gallium Maltolate Response in Glioblastoma: Comparative Analyses in 2D and 3D Models
Source: Mol Pharm. 2026 Jan 16;23(2):1072–88. doi: 10.1021/acs.molpharmaceut.5c01472 (PMC13296477; doi:10.1021/acs.molpharmaceut.5c01472)
Supplement: Supplementary file 1 [file mp5c01472_si_001.pdf]

Title: Culture Dimensionality Modulates Gallium Maltolate Response in Glioblastoma: Comparative Analyses in 2D and 3D Models

Supplementary Materials

Author names: Paulina Szeliska[1], Karol Jaroch[1], Weronika Wróblewska[1], Łukasz Kaźmierski[2], Małgorzata Maj[2], Barbara Bojko[1,]*

Affiliation:

1. Department of Pharmacodynamics and Molecular Pharmacology, Faculty of Pharmacy, Collegium Medicum, Nicolaus Copernicus University, Jurasza 2, 85-089 Bydgoszcz, Poland

2. Urology and Andrology, Department of Tissue Engineering, Collegium Medicum, Nicolaus Copernicus University, M. Curie Skłodowskiej 9, 85-094 Bydgoszcz, Poland

*Corresponding author: bbojko@cm.umk.pl

Table S1. Basic characteristics and selected genes expression of HGCC glioblastoma cells.

	**U3005**	**U3019**	**U3034**	**U3048**	**U3073**
HGCC_Subtype	Classical (CL)	Proneural (PN)	Mesenchymal (MS)	Classical (CL)	Mesenchymal (MS)
Sex	Male	Female	Male	Male	Male
Age (year)	65	82	73	77	71
Overall Survival (days)	26	117	539	279	481
Vital Status	DEAD	DEAD	DEAD	DEAD	DEAD
Diagnosis	Glioblastoma	Glioblastoma	Glioblastoma	Glioblastoma	Glioblastoma
WHO Grade	IV	IV	IV	IV	IV
NOTCH1	9.996815	10.61222	9.398689	10.70968	8.885497
TOP2A	11.01101	10.51328	11.15563	10.99726	10.34002
CD44	10.01567	9.935777	10.83175	8.009012	11.25937
PTPRCAP	8.232547	8.75818	8.020247	8.348685	8.470923
CDK4	12.01458	12.42011	11.92445	11.76847	11.80598
HNRNPUL1	11.78389	11.44992	11.44588	12.04385	11.11363
PDGFRA	10.9287	7.913807	10.65414	9.322822	9.512683
TFR2	7.596798	7.518998	6.786113	7.373756	6.925768
TFRC	8.912609	8.665308	11.06026	9.054679	11.26694
MGMT	6.996465	7.888252	7.836283	7.375181	6.918314
EGFR	9.64811	6.98694	10.34332	11.24844	9.411814

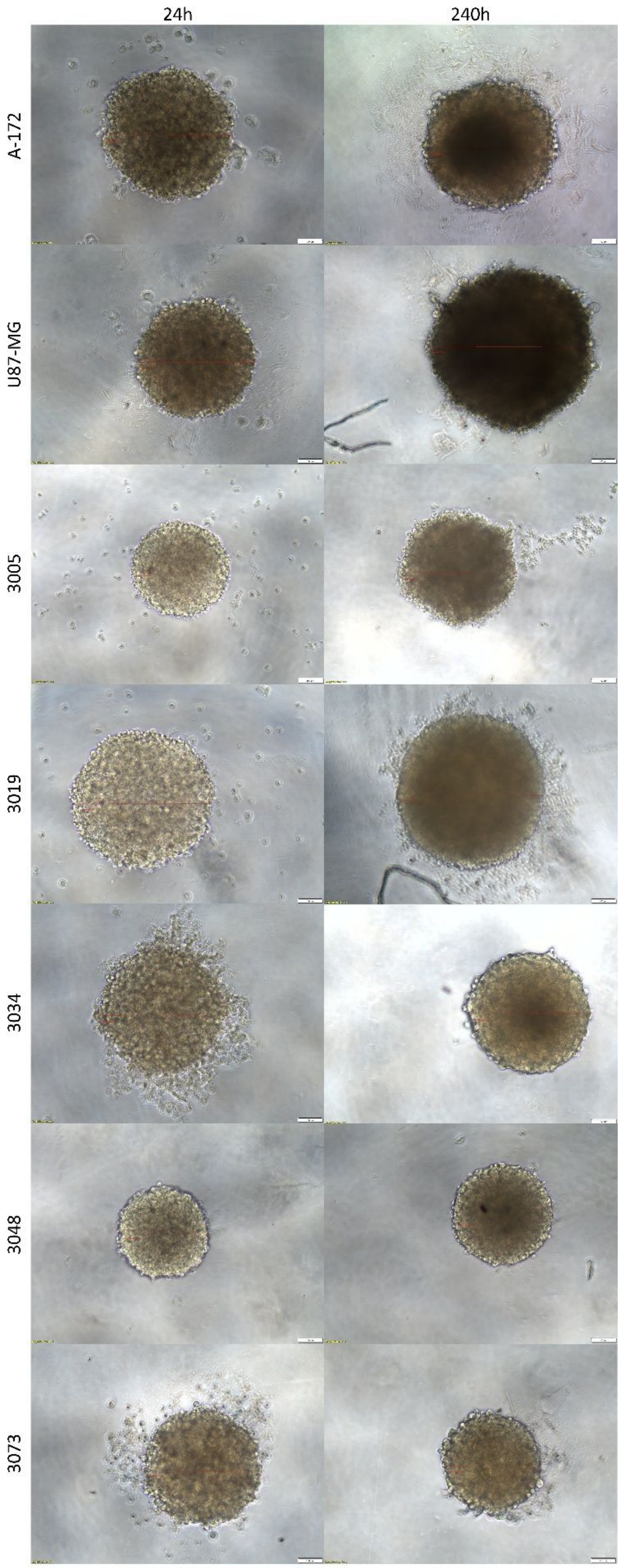

Figure S1. Photos of speroids 24h (1 day) after formation and 240h (10 days) after formation.

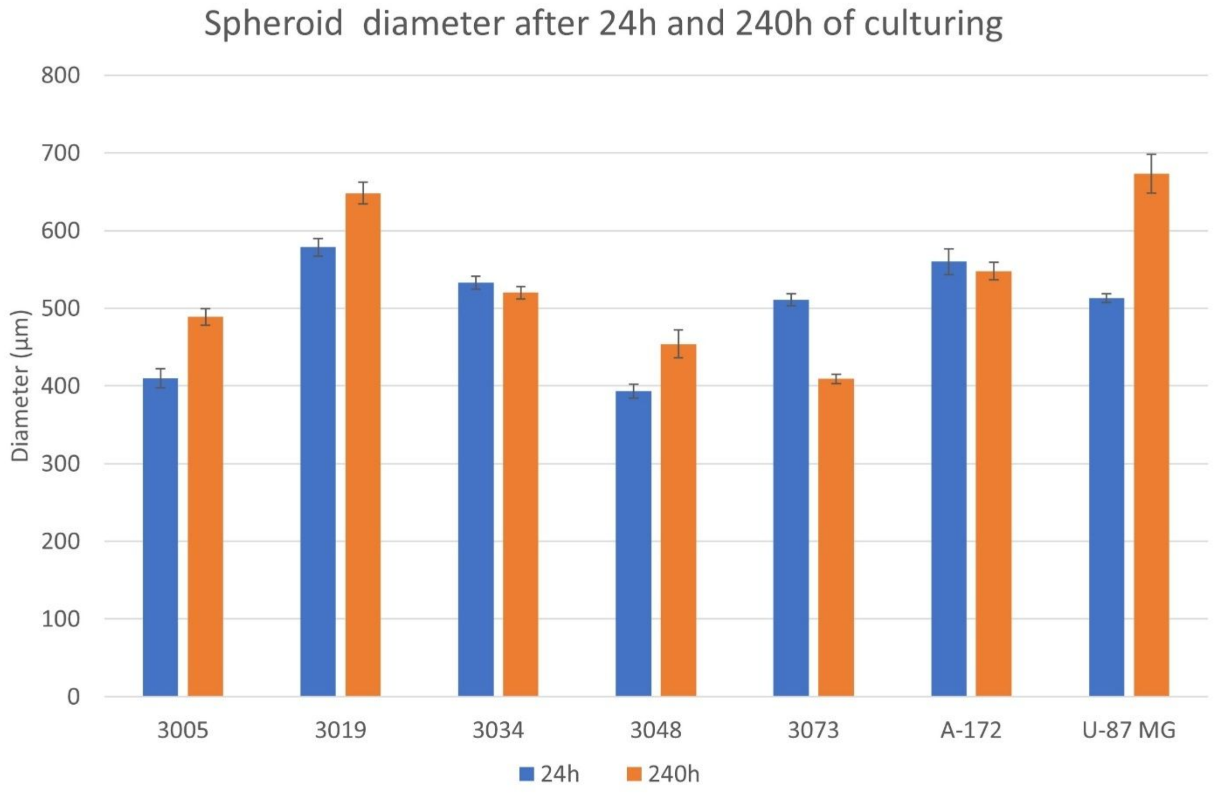

Figure S2. The spheroid diameter (µm) after the first 24h of incubation and 240h (10 days) after incubation (six replicates). The error bars represent relative standard deviation (RSD).

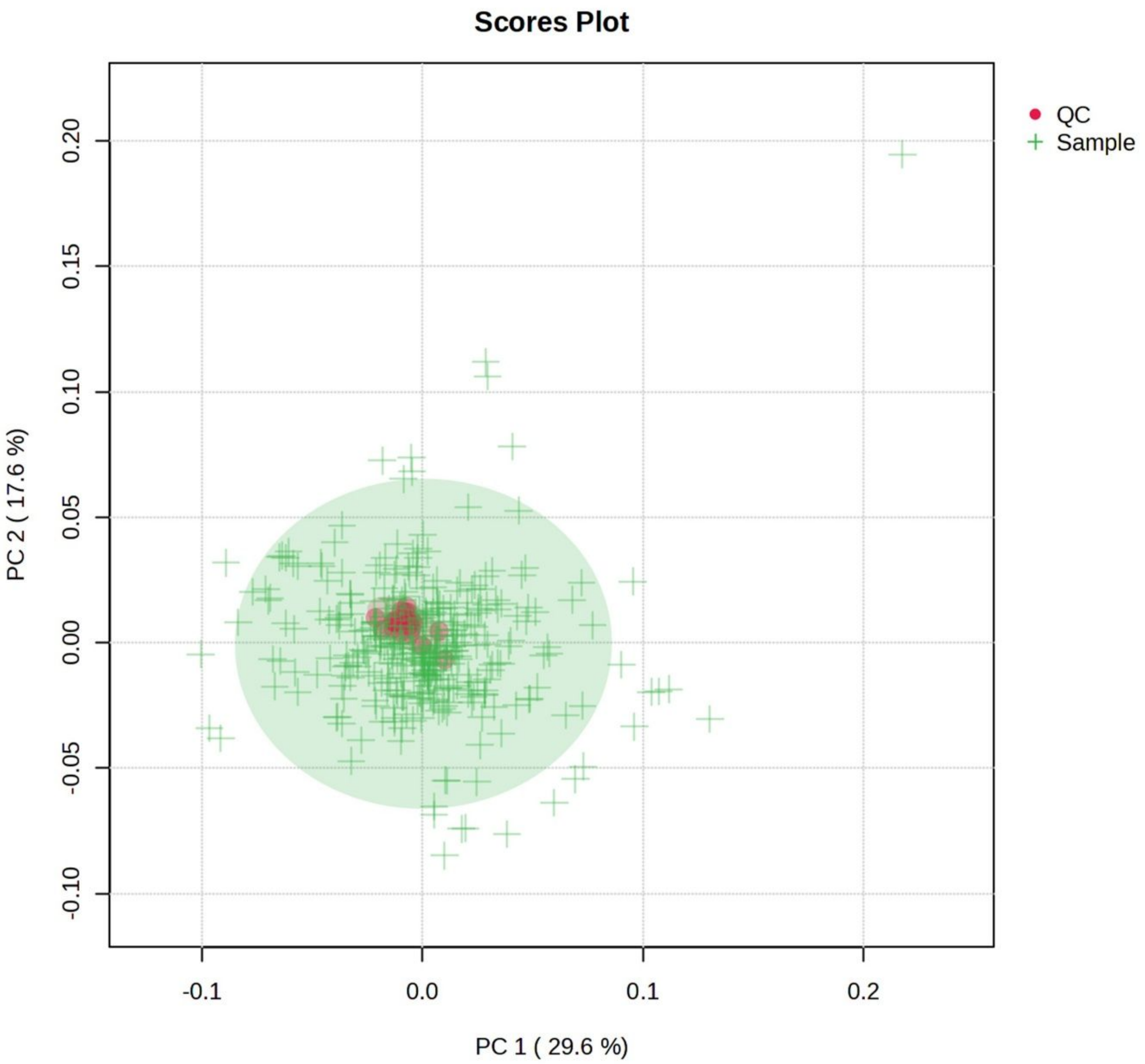

Figure S3. Principal component analysis (PCA) score plots of all analyzed samples and extraction quality control (QC) samples.

A-172

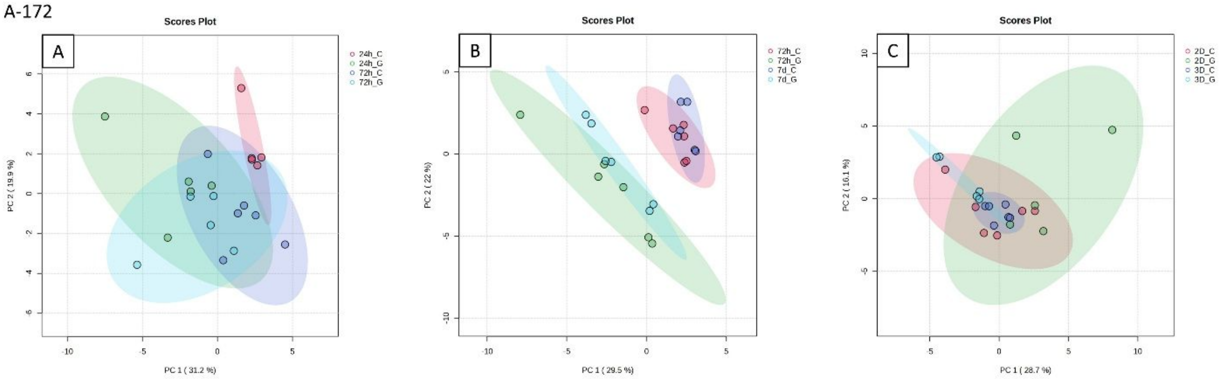

U-87 MG

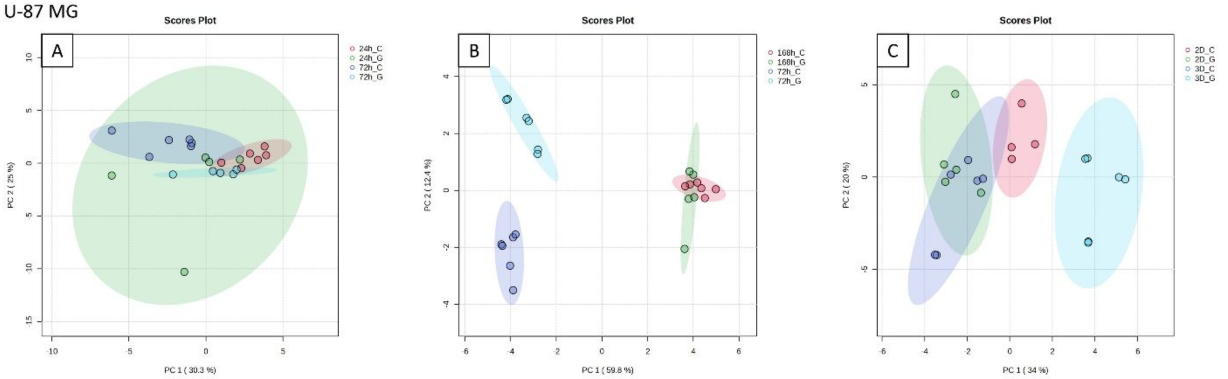

3005

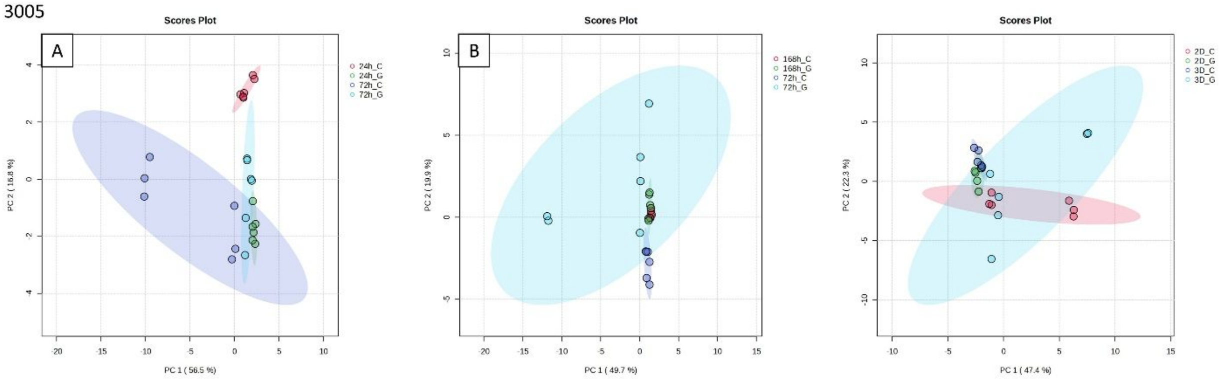

3019

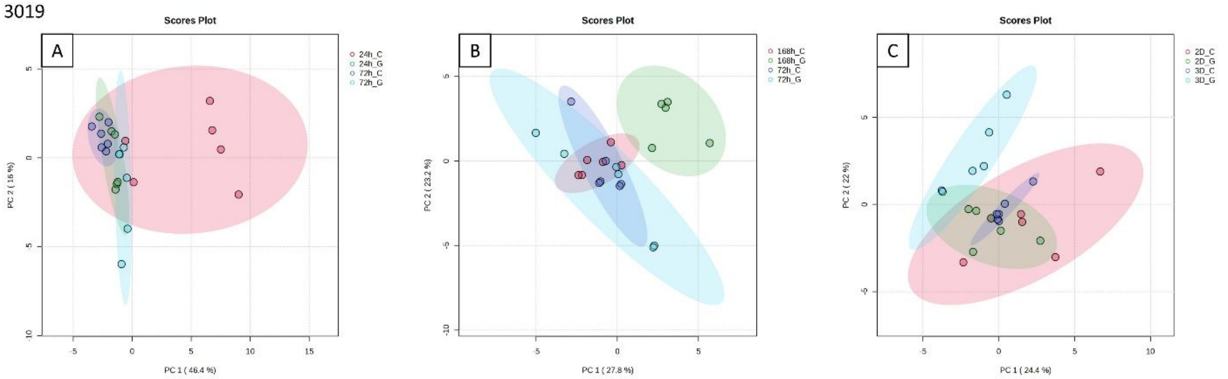

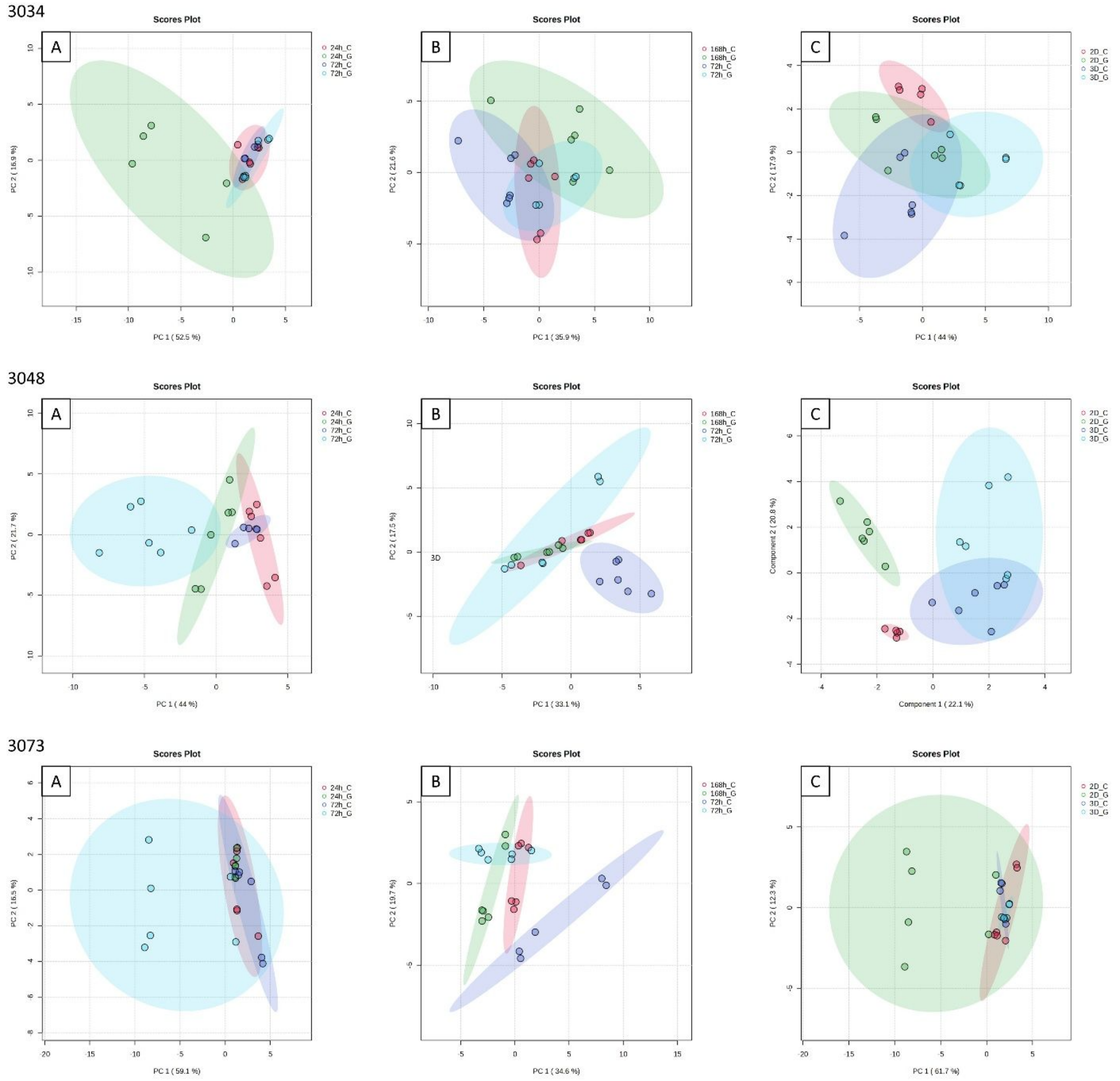

Figure S4. PCA score plots showing separation of all cell lines in A) 2D 24h vs 72h, B) 3D 72h vs 168h, C) 72h 2D vs 3D (n=6).

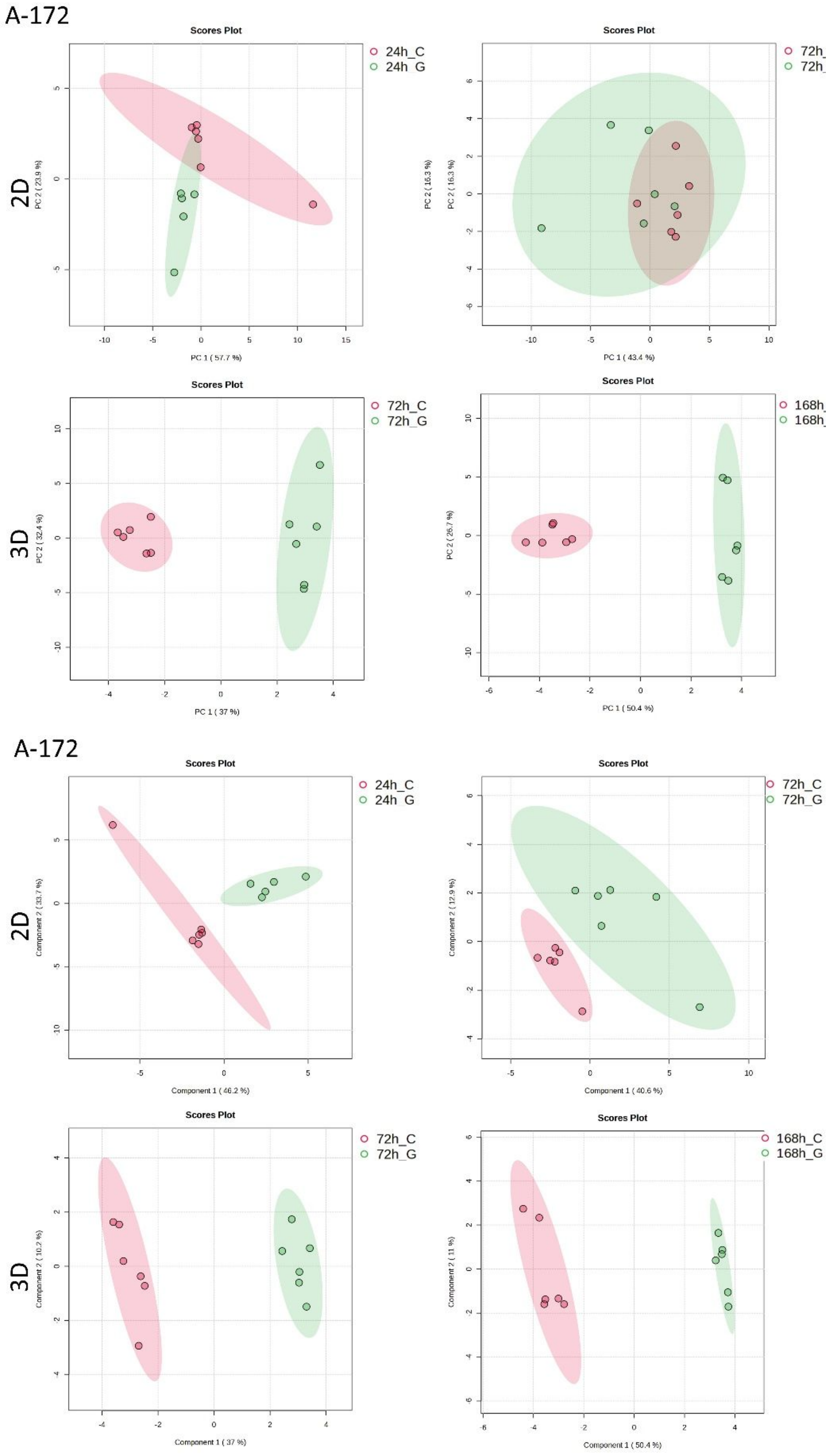

Figure S5. PCA (top) and PLS-DA scrore plots showing separations of A-172 cell line in 2D and 3D, treated (G) and untreated (C).

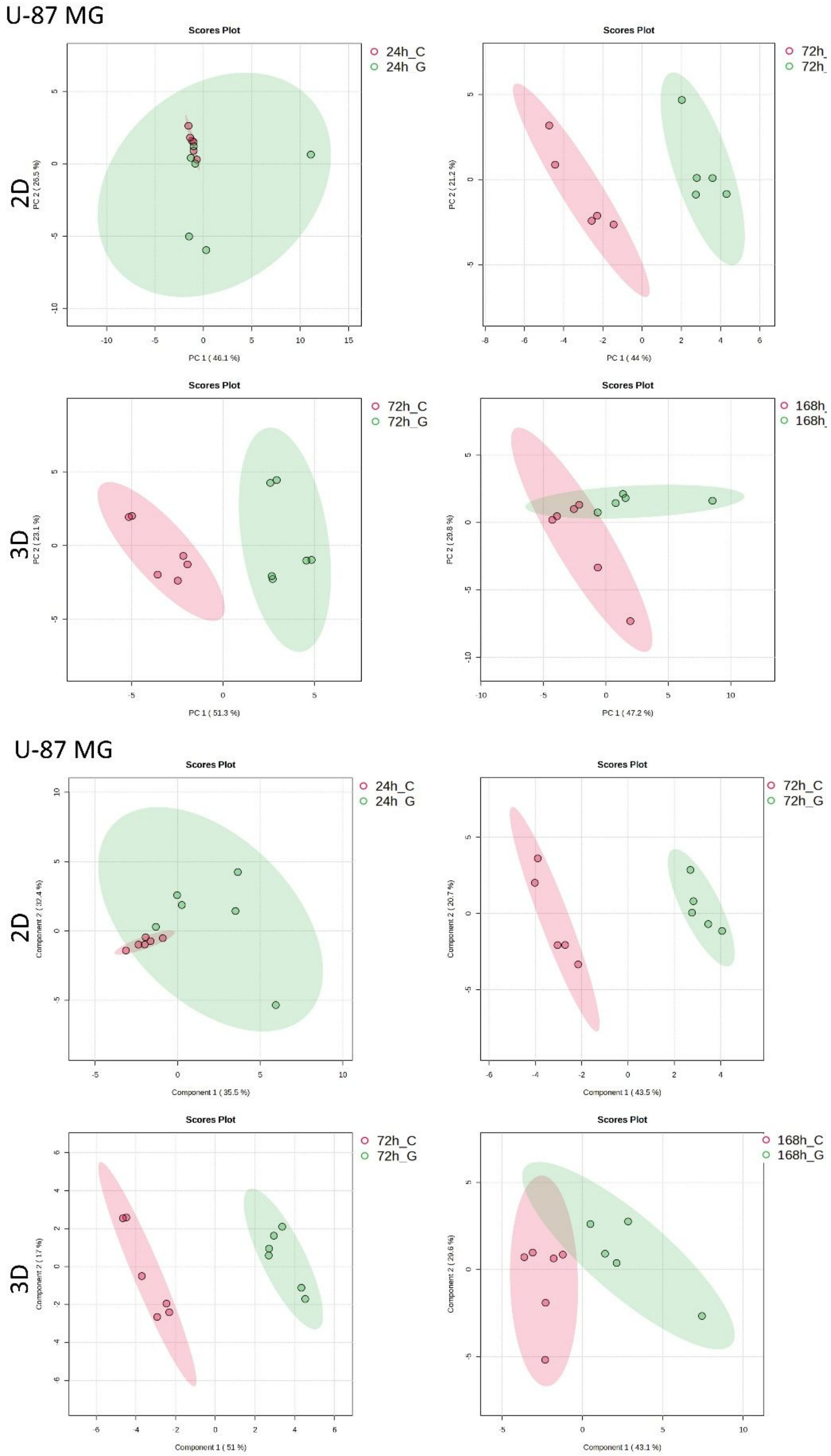

Figure S6. PCA (top) and PLS-DA scrore plots showing separations of U-87 MG cell line in 2D and 3D, treated (G) and untreated (C).

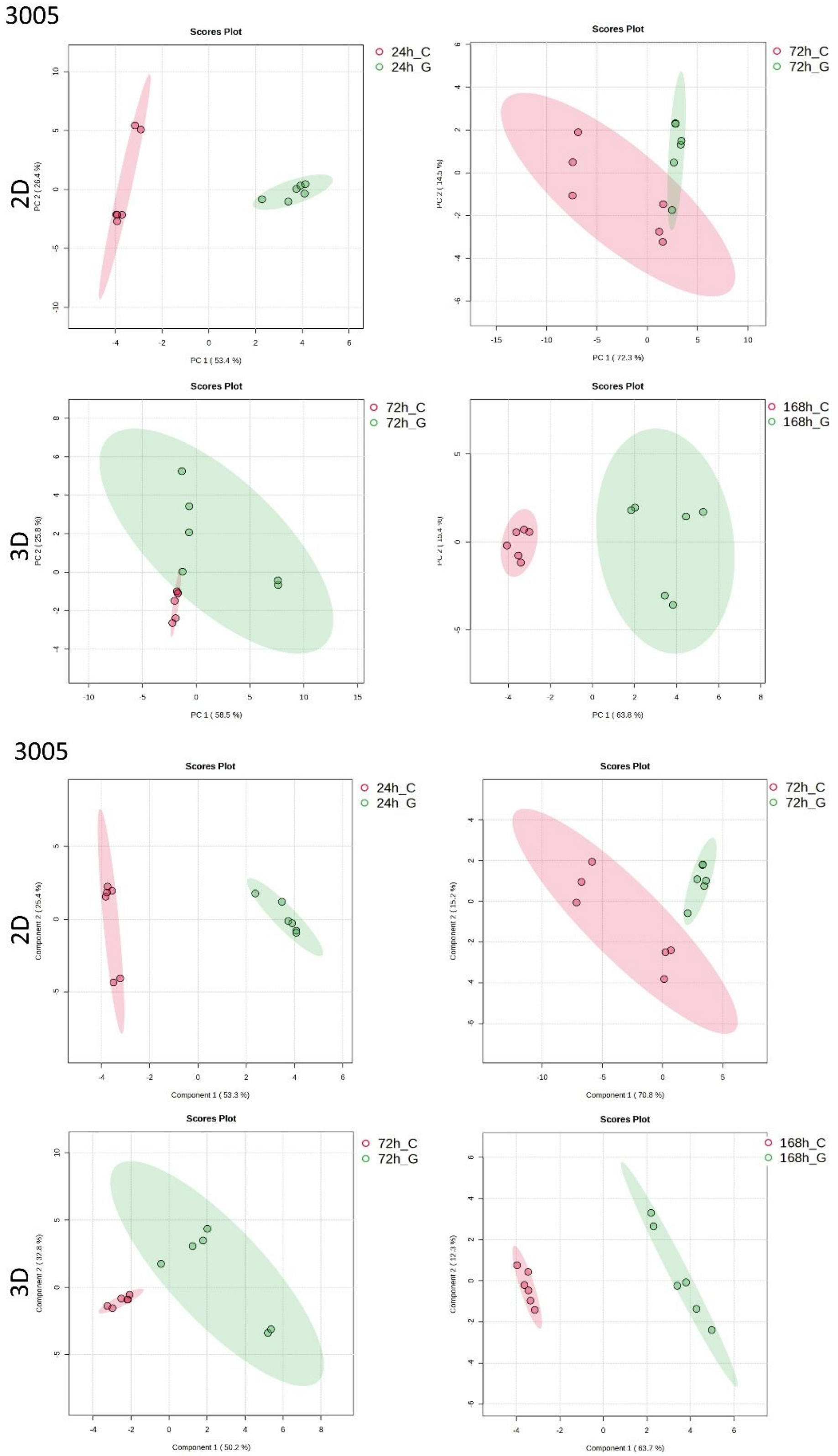

Figure S7. PCA (top) and PLS-DA scrore plots showing separations of 3005 MG cell line in 2D and 3D, treated (G) and untreated (C).

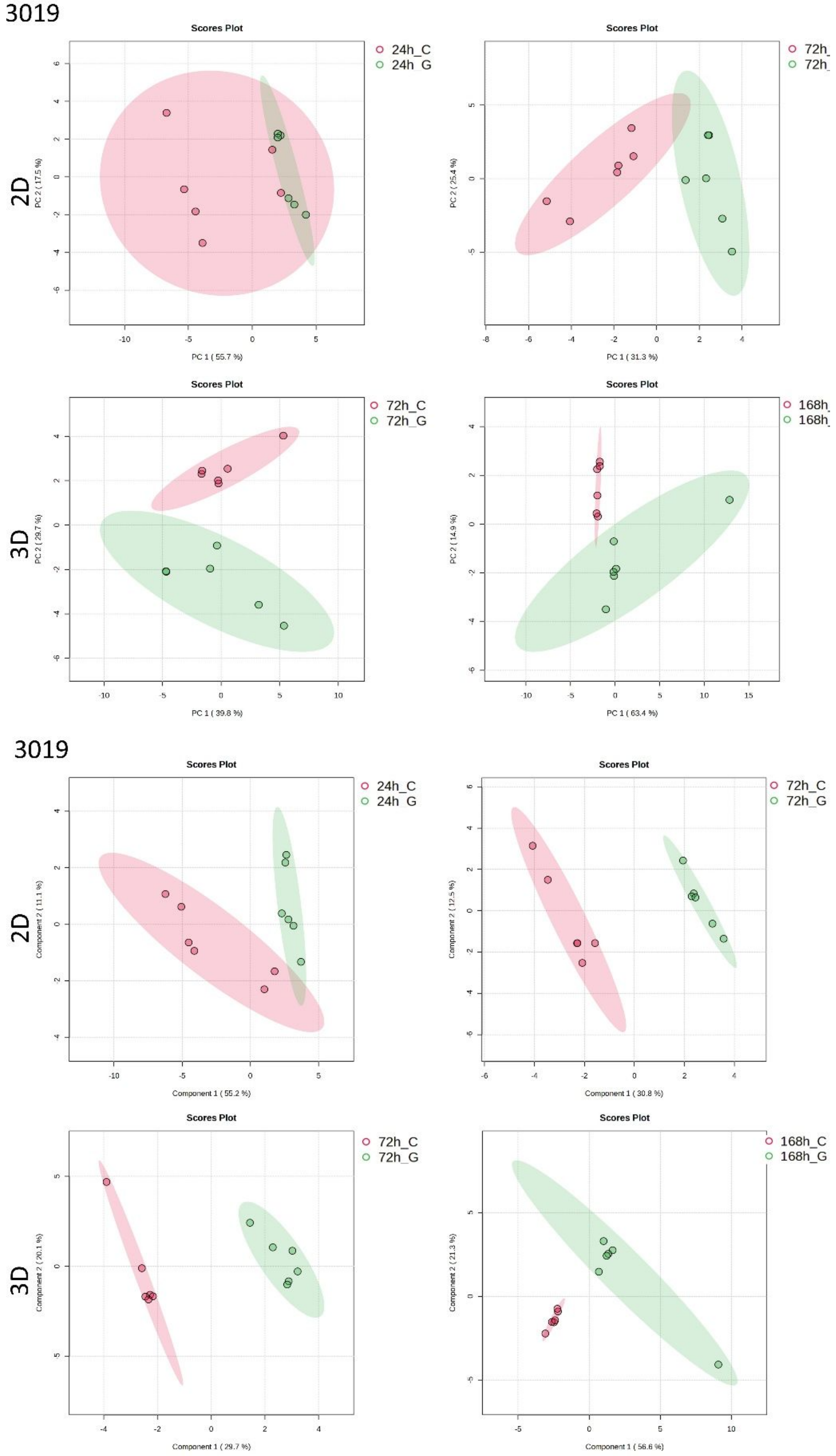

Figure S8. PCA (top) and PLS-DA scrore plots showing separations of 3019 MG cell line in 2D and 3D, treated (G) and untreated (C).

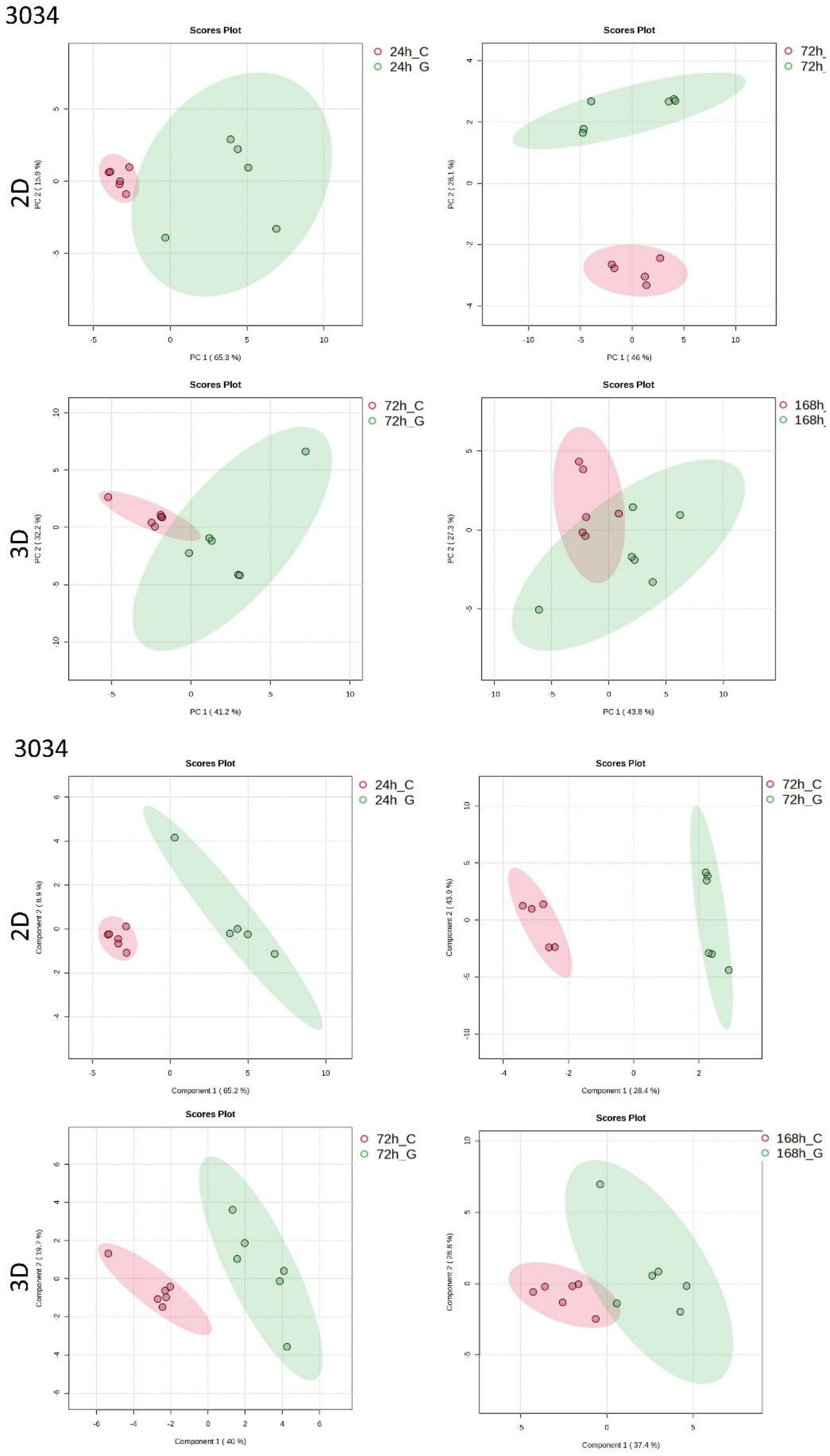

Figure S9. PCA (top) and PLS-DA scrore plots showing separations of 3034 MG cell line in 2D and 3D, treated (G) and untreated (C).

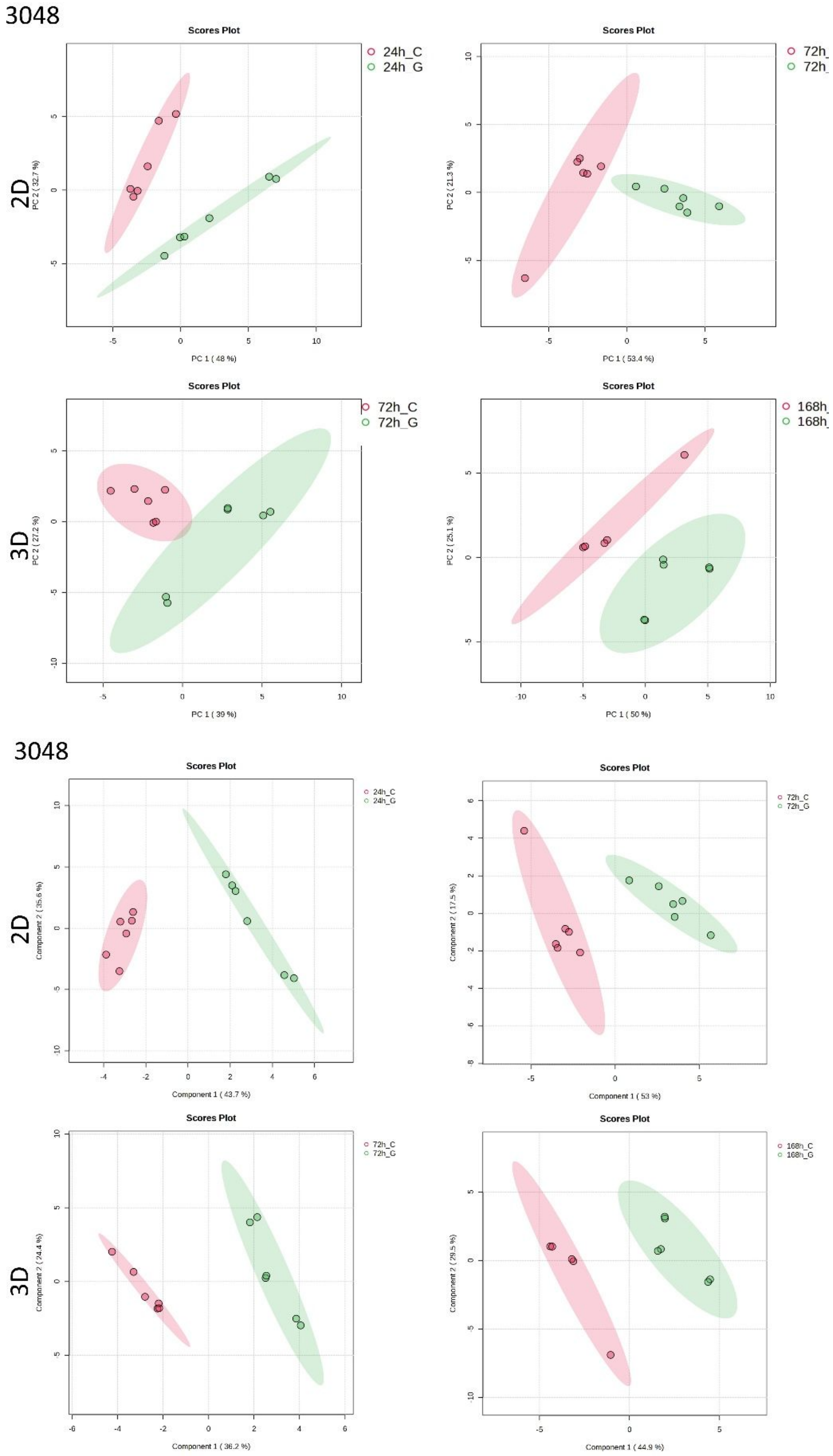

Figure S10. PCA (top) and PLS-DA scrore plots showing separations of 3048 MG cell line in 2D and 3D, treated (G) and untreated (C).

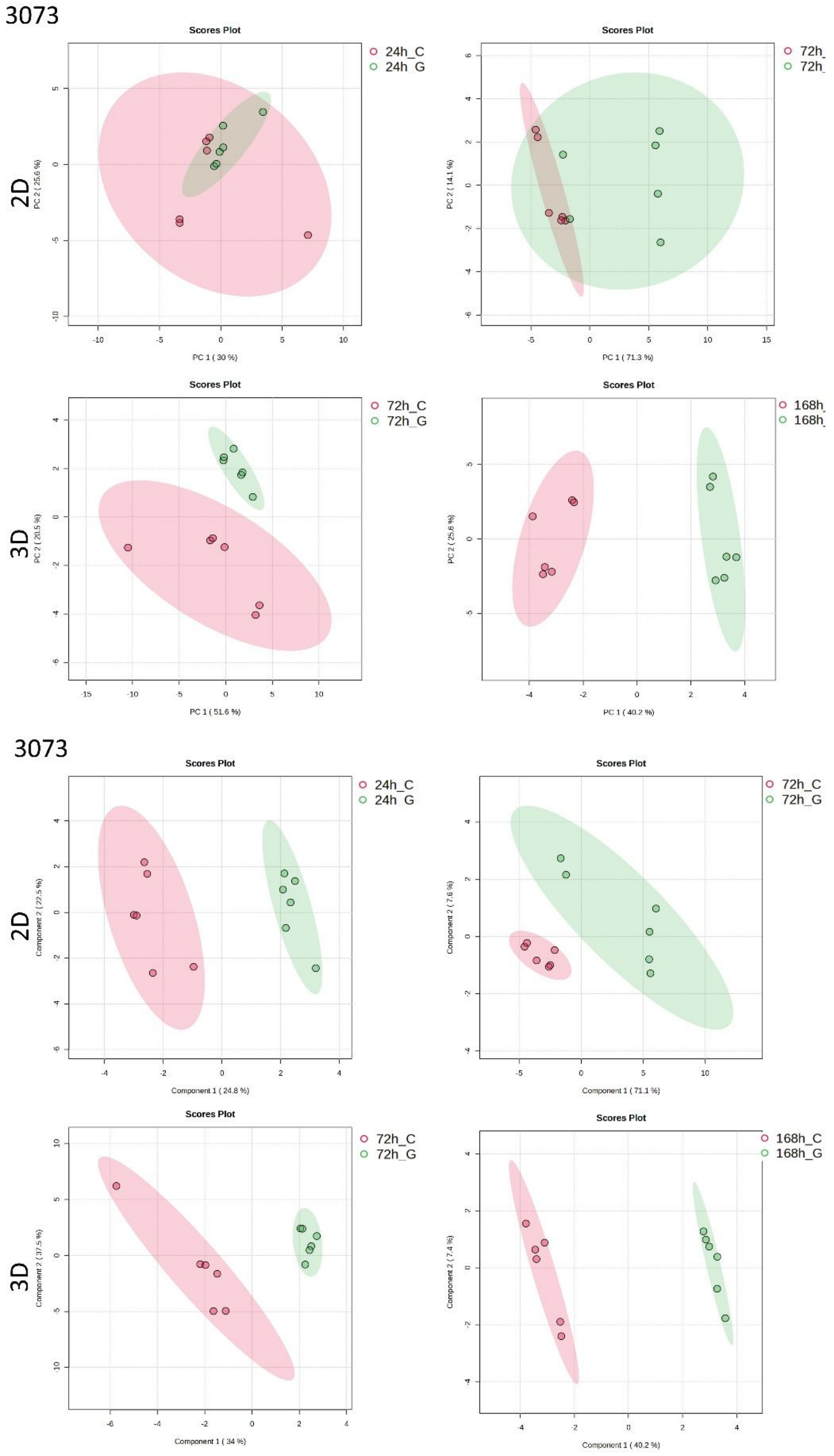

Figure S11. PCA (top) and PLS-DA scrore plots showing separations of 3073 MG cell line in 2D and 3D, treated (G) and untreated (C).

Table S2. Cross-validated PLS-DA performance across GBM models.

Cell Line	Format	Time	Comparison	Best Components	Accuracy	Q2	R2
3005	2D	24h_72h	2D_24h_72h	4	0.96	0.65474	0.84238
	2D	24h	2D_24h	3	1	0.97996	0.99671
	2D_3D	72h	2D_3D_72h	3	0.70667	0.65796	0.88375
	2D	72h	2D_72h	4	1	0.89178	0.98585
	3D	168h	3D_168h	4	1	0.98378	0.99883
	3D	72h_168h	3D_72h_168h	3	0.79333	0.68473	0.90763
	3D	72h	3D_72h	6	0.95	0.89893	0.99977
3019	2D	24h_72h	2D_24h_72h	2	0.62	0.35086	0.57689
	2D	24h	2D_24h	4	0.8	0.69851	0.94982
	2D_3D	72h	2D_3D_72h	3	0.77143	0.87795	0.92452
	2D	72h	2D_72h	3	1	0.97771	0.99513
	3D	168h	3D_168h	2	1	0.7575	0.96044
	3D	72h_168h	3D_72h_168h	2	0.47333	0.19839	0.53053
	3D	72h	3D_72h	5	1	0.97565	0.99942
3034	2D	24h_72h	2D_24h_72h	3	0.82	0.91722	0.96921
	2D	24h	2D_24h	5	1	0.92275	0.99257
	2D_3D	72h	2D_3D_72h	4	0.82	0.82507	0.94573
	2D	72h	2D_72h	2	0.83333	-0.30939	0.94595
	3D	168h	3D_168h	1	0.9	0.39949	0.71562
	3D	72h_168h	3D_72h_168h	2	0.475	0.76311	0.87437
	3D	72h	3D_72h	3	1	0.90148	0.97777
3048	2D	24h_72h	2D_24h_72h	3	0.73333	0.74137	0.86342
	2D	24h	2D_24h	3	1	0.95536	0.99102
	2D_3D	72h	2D_3D_72h	4	0.93333	0.91498	0.96714
	2D	72h	2D_72h	4	1	0.95217	0.99529
	3D	168h	3D_168h	5	1	0.98214	0.99866
	3D	72h_168h	3D_72h_168h	2	0.56333	0.57985	0.86422
	3D	72h	3D_72h	5	1	0.98625	0.99899
3073	2D	24h_72h	2D_24h_72h	5	0.83	0.80533	0.94726
	2D	24h	2D_24h	5	0.93333	0.81003	0.99811
	2D_3D	72h	2D_3D_72h	4	0.82	0.57748	0.85255
	2D	72h	2D_72h	5	1	0.93482	0.99616
	3D	168h	3D_168h	4	1	0.98378	0.99883
	3D	72h_168h	3D_72h_168h	3	0.88	0.65929	0.87341
	3D	72h	3D_72h	5	1	0.96631	0.99908
A-172	2D	24h	2D_24h	5	1	0.93118	0.99647

	2D_3D	72h	2D_3D_72h	3	0.64	0.49049	0.87458
	2D	72h	2D_72h	4	0.8	0.58358	0.95365
	3D	168h	3D_168h	2	1	0.98727	0.99521
	3D	72h+168 h	3D_72h+168h	4	1	0.95995	0.99722
	3D	72h_168 h	3D_72h_168h	5	0.96	0.92071	0.97342
	2D	24h_72h	2D_24h_72h	4	0.86	0.76004	0.9672
U-87 MG	2D	24h_72h	2D_24h_72h	4	0.83	0.68205	0.93221
	2D	24h	2D_24h	1	0.95	-0.35231	0.75069
	2D_3D	72h	2D_3D_72h	5	1	0.74933	0.98245
	2D	72h	2D_72h	3	0.83333	0.40482	0.84569
	3D	168h	3D_168h	1	0.9	0.39167	0.69546
	3D	72h_168 h	3D_72h_168h	2	0.78	0.91827	0.95246
	3D	72h	3D_72h	5	1	0.98934	0.99951

Table S3. Comprehensive metabolite panel by cell line (3005, 3019, 3034, 3048, 3073, A-172, U-87 MG): VIP, FDR, stars (* = p ≤ 0.05, ** = p ≤ 0.01).

Cell Line	Format	Time	Condition	Metabolite	VIP	FDR	stars
3005	2D	24h	2D_24h	Allantoin	1.2467	0.008434	**
	3D	168h	3D_168h	Allantoin	1.0036	0.006815	**
	2D	24h	2D_24h	Choline	1.3072	0.008434	**
	3D	168h	3D_168h	Choline	1.1882	0.003968	**
	2D	24h	2D_24h	Cysteine	1.0935	0.006494	**
	2D	72h	2D_72h	Cysteine	1.299	0.011688	*
	3D	168h	3D_168h	Cysteine	1.1649	0.003968	**
	3D	72h	3D_72h	Cysteine	1.5496	0.02381	*
	2D	24h	2D_24h	Glutamicacid	1.0655	0.006494	**
	2D	72h	2D_72h	Glutamine	1.3858	0.011688	*
	3D	168h	3D_168h	Glutamine	1.254	0.003968	**
	3D	72h	3D_72h	Glutamine	1.5778	0.015873	*
	3D	168h	3D_168h	Histidine	1.2612	0.003968	**
	2D	72h	2D_72h	Isoleucine	1.2834	0.011688	*
	2D	24h	2D_24h	Lysine	1.2104	0.006494	**
	3D	168h	3D_168h	Lysine	1.1372	0.003968	**
	2D	72h	2D_72h	Methionine	1.1695	0.02904	*
	3D	168h	3D_168h	Methionine	1.09	0.003968	**
	2D	24h	2D_24h	Niacinamide	1.1203	0.008434	**
	2D	72h	2D_72h	Niacinamide	1.3112	0.014994	*
	3D	168h	3D_168h	Niacinamide	1.0173	0.003968	**
	3D	72h	3D_72h	Niacinamide	1.6055	0.015873	*
	3D	72h	3D_72h	Ornithine	1.4831	0.031746	*
	2D	24h	2D_24h	Phenylalanine	1.1686	0.006494	**
	2D	72h	2D_72h	Phenylalanine	1.2333	0.011688	*
	3D	168h	3D_168h	Phenylalanine	1.2398	0.003968	**
	2D	24h	2D_24h	Proline	1.3449	0.006494	**
	3D	168h	3D_168h	Proline	1.1253	0.003968	**
	2D	72h	2D_72h	Serine	1.126	0.025213	*
	3D	168h	3D_168h	Serine	1.0912	0.003968	**
	2D	24h	2D_24h	Threonine	1.0547	0.013751	*
	2D	24h	2D_24h	Tryptophan	1.258	0.008434	**
	2D	72h	2D_72h	Tryptophan	1.2273	0.014994	*
	3D	168h	3D_168h	Tryptophan	1.0225	0.006815	**
	2D	24h	2D_24h	Tyrosine	1.2133	0.006494	**
	2D	72h	2D_72h	Tyrosine	1.0667	0.023377	*
	2D	24h	2D_24h	Uracil	1.2965	0.008434	**
	2D	72h	2D_72h	Uracil	1.3756	0.011688	*
	3D	168h	3D_168h	Uracil	1.2685	0.003968	**

	3D	72h	3D_72h	Uracil	1.4161	0.015873	*
	2D	24h	2D_24h	Valine	1.2714	0.006494	**
3019	2D	72h	2D_72h	Allantoin	1.1145	0.019481	*
	3D	72h	3D_72h	Allantoin	1.0505	0.01461	*
	3D	72h	3D_72h	Glutamicacid	1.3095	0.019481	*
	3D	72h	3D_72h	Histidine	1.5642	0.01461	*
	2D	24h	2D_24h	Lysine	1.3021	0.040909	*
	3D	168h	3D_168h	Lysine	1.0544	0.007305	**
	2D	24h	2D_24h	Methionine	1.4629	0.011688	*
	3D	168h	3D_168h	Methionine	1.5066	0.007305	**
	2D	24h	2D_24h	Niacinamide	1.3656	0.027053	*
	2D	24h	2D_24h	Ornithine	1.3793	0.040909	*
	2D	24h	2D_24h	Proline	1.4157	0.019278	*
	3D	168h	3D_168h	Serine	1.0485	0.007305	**
	2D	72h	2D_72h	Tryptophan	1.631	0.02699	*
	2D	24h	2D_24h	Tyrosine	1.3967	0.011688	*
	3D	168h	3D_168h	Tyrosine	1.4581	0.007305	**
	2D	24h	2D_24h	Uracil	1.5132	0.011688	*
	2D	72h	2D_72h	Uracil	1.8454	0.02699	*
	3D	72h	3D_72h	Uracil	1.8903	0.01461	*
	2D	24h	2D_24h	Valine	1.3542	0.011688	*
3034	2D	24h	2D_24h	Adenosine	1.1594	0.011688	*
	3D	72h	3D_72h	Allantoin	1.6015	0.00974	**
	2D	24h	2D_24h	Glutamicacid	1.1628	0.011688	*
	3D	72h	3D_72h	Glutamine	1.4951	0.00974	**
	2D	24h	2D_24h	Histidine	1.3424	0.011688	*
	2D	24h	2D_24h	Leucine	1.2506	0.011688	*
	2D	24h	2D_24h	Lysine	1.191	0.011688	*
	3D	72h	3D_72h	Methionine	1.5239	0.00974	**
	3D	72h	3D_72h	Niacinamide	1.4326	0.00974	**
	2D	24h	2D_24h	Proline	1.2001	0.019481	*
	2D	24h	2D_24h	Tryptophan	1.2562	0.011688	*
	3D	168h	3D_168h	Tryptophan	1.3983	0.044983	*
	3D	72h	3D_72h	Tryptophan	1.2669	0.033395	*
	3D	168h	3D_168h	Tyrosine	1.6383	0.044983	*
	2D	24h	2D_24h	Uracil	1.2789	0.011688	*
	2D	72h	2D_72h	Uracil	1.8786	0.029221	*
	3D	72h	3D_72h	Uracil	1.6662	0.00974	**
	2D	24h	2D_24h	Valine	1.1976	0.011688	*
3048	2D	24h	2D_24h	Allantoin	1.3721	0.007305	**
	2D	72h	2D_72h	Allantoin	1.2524	0.008349	**
	3D	168h	3D_168h	Allantoin	1.6831	0.015004	*
	2D	24h	2D_24h	Cysteine	1.0207	0.007305	**

	2D	72h	2D_72h	Glutamicacid	1.1458	0.005313	**
	2D	72h	2D_72h	Glutamine	1.3019	0.008245	**
	3D	72h	3D_72h	Glutamine	1.5045	0.00974	**
	2D	24h	2D_24h	Histidine	1.2581	0.007305	**
	2D	24h	2D_24h	Isoleucine	1.1973	0.007305	**
	2D	72h	2D_72h	Isoleucine	1.2135	0.008349	**
	2D	24h	2D_24h	Leucine	1.1596	0.023377	*
	2D	72h	2D_72h	Leucine	1.0581	0.005313	**
	3D	168h	3D_168h	Leucine	1.3655	0.033395	*
	2D	72h	2D_72h	Lysine	1.3763	0.005313	**
	2D	72h	2D_72h	Methionine	1.4176	0.005313	**
	3D	168h	3D_168h	Methionine	1.4213	0.01461	*
	3D	72h	3D_72h	Niacinamide	1.4832	0.00974	**
	3D	168h	3D_168h	Ornithine	1.2558	0.033395	*
	2D	72h	2D_72h	Proline	1.237	0.005313	**
	3D	72h	3D_72h	Proline	1.211	0.045455	*
	2D	24h	2D_24h	Serine	1.381	0.007305	**
	2D	72h	2D_72h	Serine	1.2242	0.005313	**
	2D	72h	2D_72h	Tryptophan	1.3436	0.005313	**
	3D	168h	3D_168h	Tryptophan	1.2142	0.01461	*
	3D	72h	3D_72h	Tryptophan	1.3644	0.00974	**
	2D	24h	2D_24h	Tyrosine	1.3969	0.007305	**
	3D	72h	3D_72h	Tyrosine	1.273	0.00974	**
	2D	72h	2D_72h	Uracil	1.2202	0.005313	**
	3D	168h	3D_168h	Uracil	1.4293	0.01461	*
	3D	72h	3D_72h	Uracil	1.6387	0.00974	**
	2D	24h	2D_24h	Valine	1.3179	0.007305	**
	3D	168h	3D_168h	Valine	1.4871	0.01461	*
	3D	72h	3D_72h	Valine	1.2804	0.045455	*
3073	3D	168h	3D_168h	Adenosine	1.0458	0.046753	*
	3D	168h	3D_168h	Allantoin	1.1043	0.034091	*
	3D	72h	3D_72h	Allantoin	1.6839	0.03221	*
	2D	72h	2D_72h	Cysteine	1.1962	0.021251	*
	3D	168h	3D_168h	Cysteine	1.4669	0.008349	**
	2D	24h	2D_24h	Glutamine	1.4927	0.029221	*
	2D	72h	2D_72h	Glutamine	1.1767	0.021251	*
	3D	168h	3D_168h	Histidine	1.5285	0.008349	**
	2D	72h	2D_72h	Isoleucine	1.2023	0.008349	**
	3D	168h	3D_168h	Isoleucine	1.1648	0.012987	*
	3D	168h	3D_168h	Lysine	1.412	0.008349	**
	2D	72h	2D_72h	Methionine	1.1904	0.008349	**
	3D	72h	3D_72h	Methionine	1.3647	0.046753	*
	2D	72h	2D_72h	Niacinamide	1.3236	0.008349	**

	2D	72h	2D_72h	Phenylalanine	1.1759	0.008349	**
	3D	168h	3D_168h	Phenylalanine	1.5349	0.008349	**
	2D	24h	2D_24h	Proline	1.8999	0.029221	*
	3D	168h	3D_168h	Proline	1.0172	0.046753	*
	2D	72h	2D_72h	Serine	1.2129	0.01461	*
	3D	168h	3D_168h	Serine	1.3256	0.008349	**
	2D	72h	2D_72h	Threonine	1.188	0.021251	*
	3D	168h	3D_168h	Threonine	1.2329	0.008349	**
	2D	72h	2D_72h	Tryptophan	1.2397	0.008349	**
	3D	168h	3D_168h	Tryptophan	1.1327	0.046753	*
	3D	72h	3D_72h	Tryptophan	1.688	0.029221	*
	2D	72h	2D_72h	Uracil	1.4124	0.008349	**
	3D	168h	3D_168h	Uracil	1.2753	0.023377	*
	3D	72h	3D_72h	Uracil	1.7576	0.029221	*
	2D	24h	2D_24h	Valine	1.6083	0.029221	*
	3D	168h	3D_168h	Valine	1.2935	0.012987	*
	3D	72h	3D_72h	Valine	1.6379	0.03221	*
A-172	2D	24h	2D_24h	Allantoin	1.8205	0.012987	*
	3D	168h	3D_168h	Allantoin	1.4139	0.006251	**
	3D	72h	3D_72h	Allantoin	1.6185	0.008349	**
	3D	168h	3D_168h	Cysteine	1.2199	0.005313	**
	3D	72h	3D_72h	Cysteine	1.2619	0.008349	**
	3D	168h	3D_168h	Glutamine	1.3853	0.005313	**
	3D	168h	3D_168h	Histidine	1.1009	0.005313	**
	3D	168h	3D_168h	Leucine	1.2151	0.005313	**
	3D	168h	3D_168h	Lysine	1.3021	0.005313	**
	3D	72h	3D_72h	Lysine	1.4465	0.008349	**
	3D	168h	3D_168h	Methionine	1.2729	0.005313	**
	3D	72h	3D_72h	Methionine	1.3674	0.008349	**
	3D	168h	3D_168h	Niacinamide	1.0925	0.005313	**
	2D	24h	2D_24h	Ornithine	1.4682	0.012987	*
	3D	168h	3D_168h	Ornithine	1.0044	0.016698	*
	3D	72h	3D_72h	Ornithine	1.4309	0.008349	**
	3D	168h	3D_168h	Phenylalanine	1.3361	0.005313	**
	3D	168h	3D_168h	Serine	1.266	0.005313	**
	3D	72h	3D_72h	Serine	1.2128	0.008349	**
	2D	24h	2D_24h	Tryptophan	1.6864	0.012987	*
	3D	168h	3D_168h	Tyrosine	1.1438	0.008991	**
	2D	24h	2D_24h	Uracil	1.6579	0.012987	*
	3D	168h	3D_168h	Uracil	1.4003	0.005313	**
	3D	72h	3D_72h	Uracil	1.6082	0.008349	**
U87MG	3D	72h	3D_72h	Adenosine	1.2438	0.005313	**
	3D	72h	3D_72h	Allantoin	1.4314	0.006251	**

	3D	72h	3D_72h	Choline	1.1484	0.005313	**
	3D	72h	3D_72h	Glutamicacid	1.2518	0.005313	**
	3D	72h	3D_72h	Histidine	1.1785	0.005313	**
	3D	168h	3D_168h	Methionine	1.1634	0.046753	*
	3D	72h	3D_72h	Methionine	1.3087	0.005313	**
	3D	168h	3D_168h	Niacinamide	1.5357	0.029221	*
	3D	168h	3D_168h	Proline	1.5211	0.029221	*
	3D	72h	3D_72h	Serine	1.1842	0.005313	**
	3D	72h	3D_72h	Tryptophan	1.1085	0.005313	**
	3D	72h	3D_72h	Tyrosine	1.0347	0.027273	*
	3D	168h	3D_168h	Uracil	1.24	0.029221	*
	3D	72h	3D_72h	Uracil	1.4291	0.005313	**
	3D	168h	3D_168h	Valine	1.3233	0.029221	*

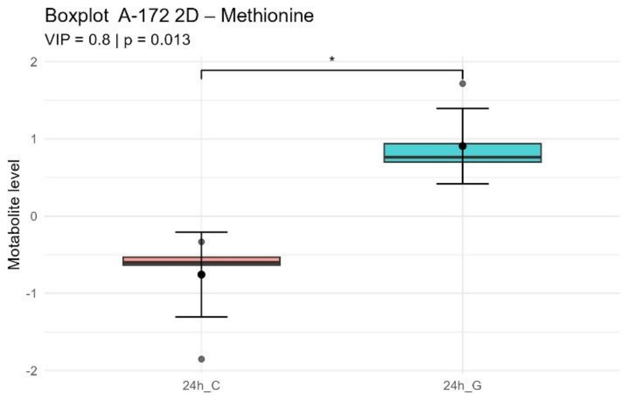
Boxplot A-172 2D – Methionine
VIP = 0.8 | p = 0.013
*
Motabolite level
24h_C
24h_G

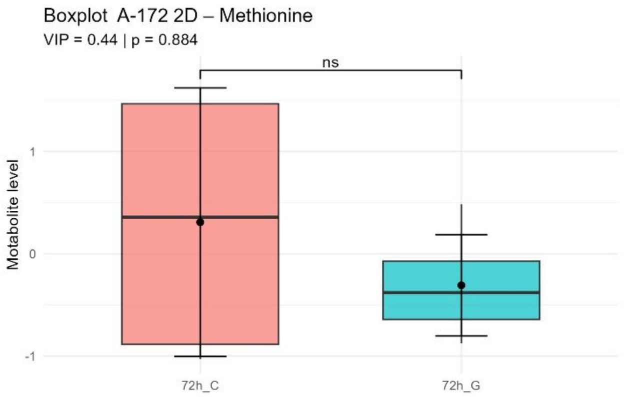
Boxplot A-172 2D – Methionine
VIP = 0.44 | p = 0.884
ns
Motabolite level
72h_C
72h_G

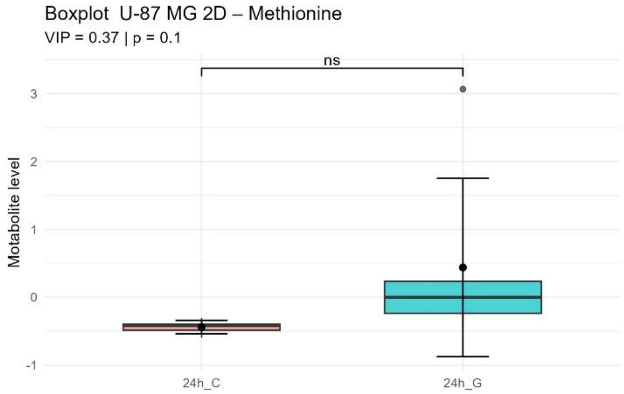
Boxplot U-87 MG 2D – Methionine
VIP = 0.37 | p = 0.1
ns
Motabolite level
24h_C
24h_G

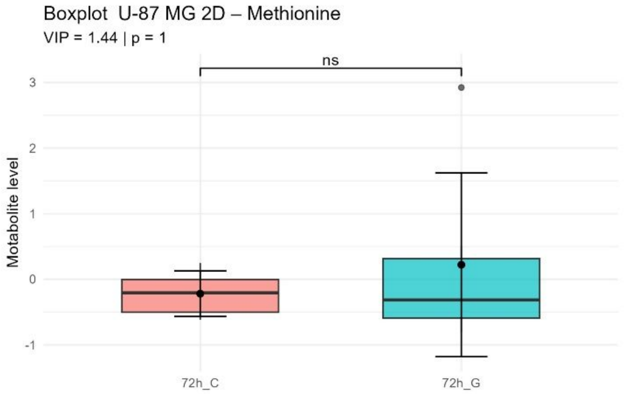
Boxplot U-87 MG 2D – Methionine
VIP = 1.44 | p = 1
ns
Motabolite level
72h_C
72h_G

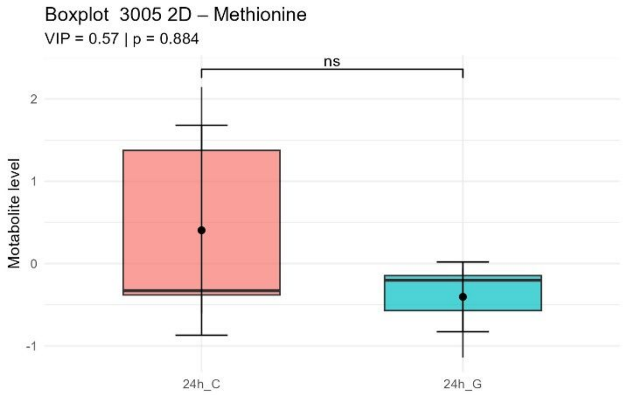
Boxplot 3005 2D – Methionine
VIP = 0.57 | p = 0.884
ns
Motabolite level
24h_C
24h_G

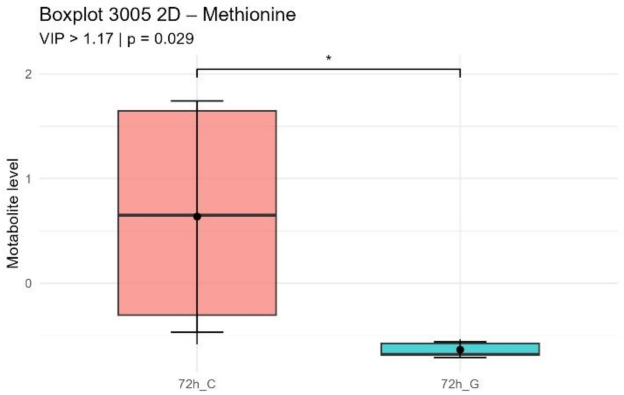
Boxplot 3005 2D – Methionine
VIP > 1.17 | p = 0.029
*
Motabolite level
72h_C
72h_G

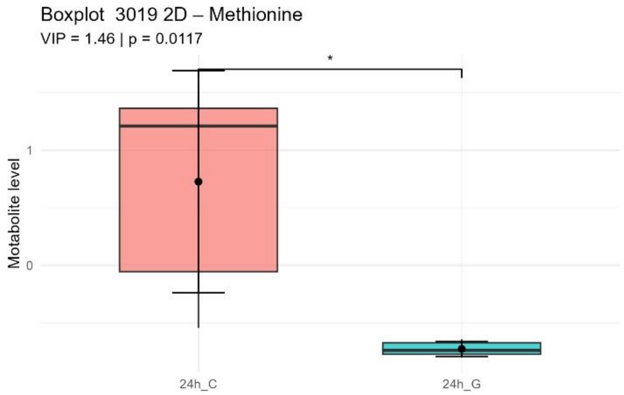
Boxplot 3019 2D – Methionine
VIP = 1.46 | p = 0.0117
*
Motabolite level
24h_C
24h_G

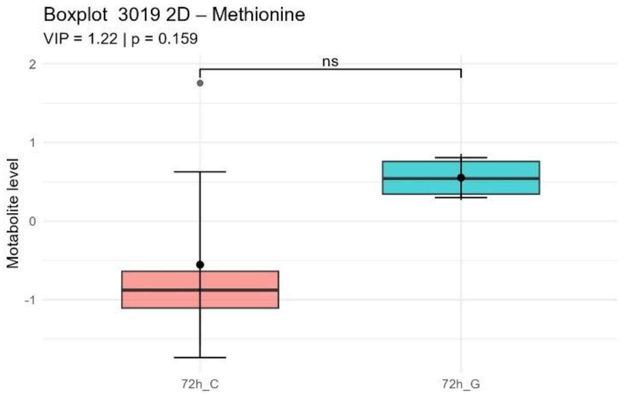
Boxplot 3019 2D – Methionine
VIP = 1.22 | p = 0.159
ns
Motabolite level
72h_C
72h_G

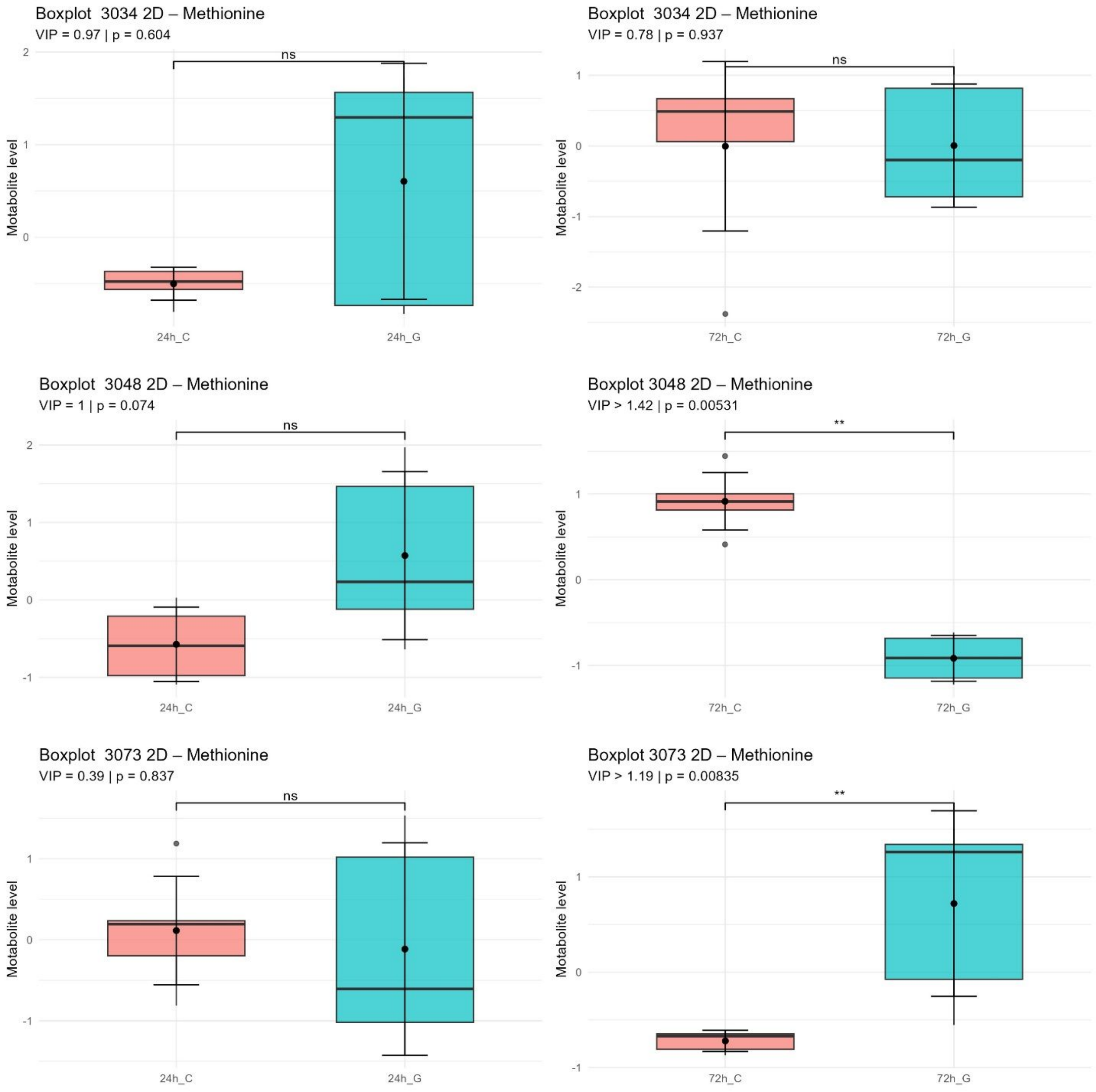

Figure S12. Change in levels of methionine between GaM treated cells and untreated control in 2D culture with VIP score and p-value (FDR). The error bars represent standard deviation (SD).

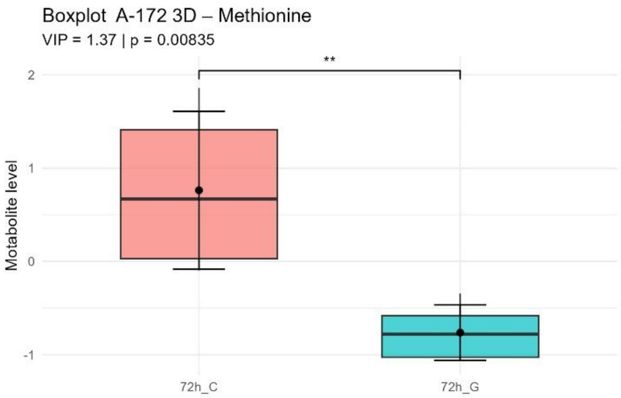
Boxplot A-172 3D – Methionine
VIP = 1.37 | p = 0.00835
**
Motabolite level
72h_C
72h_G

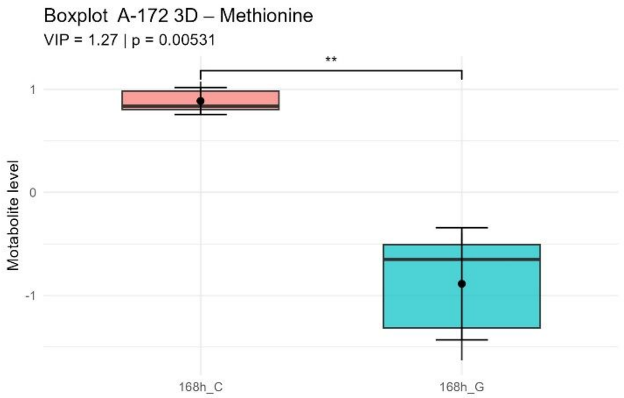
Boxplot A-172 3D – Methionine
VIP = 1.27 | p = 0.00531
**
Motabolite level
168h_C
168h_G

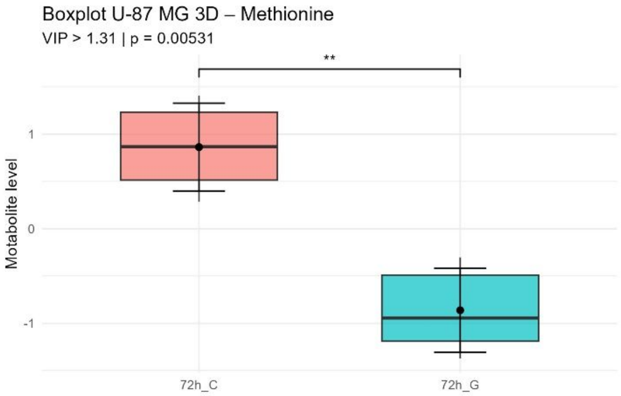
Boxplot U-87 MG 3D – Methionine
VIP > 1.31 | p = 0.00531
**
Motabolite level
72h_C
72h_G

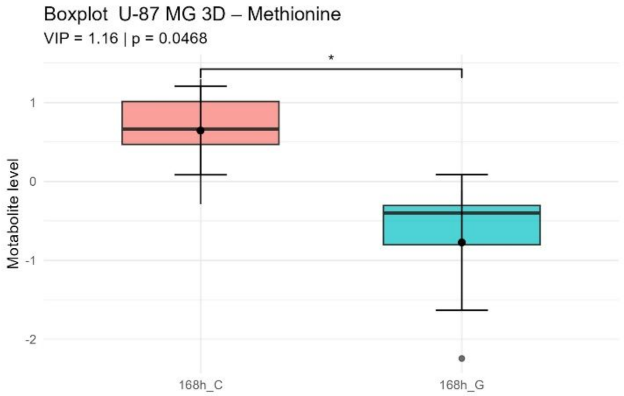
Boxplot U-87 MG 3D – Methionine
VIP = 1.16 | p = 0.0468
*
Motabolite level
168h_C
168h_G

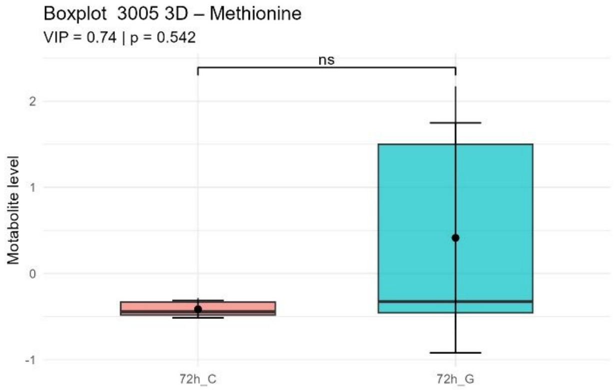
Boxplot 3005 3D – Methionine
VIP = 0.74 | p = 0.542
ns
Motabolite level
72h_C
72h_G

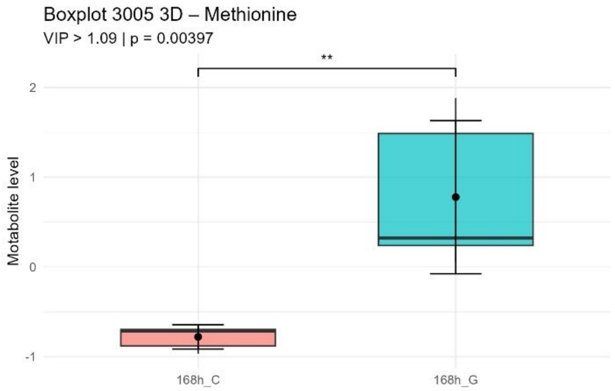
Boxplot 3005 3D – Methionine
VIP > 1.09 | p = 0.00397
**
Motabolite level
168h_C
168h_G

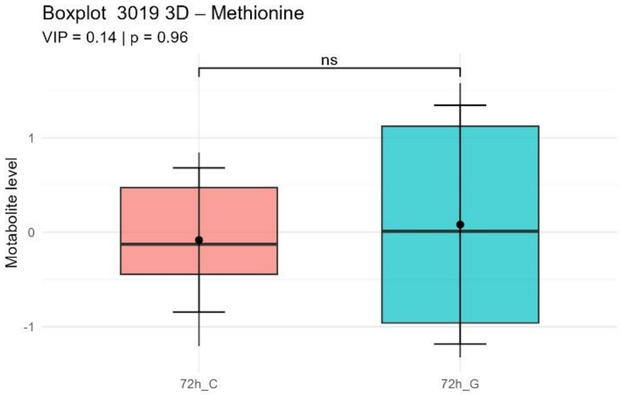
Boxplot 3019 3D – Methionine
VIP = 0.14 | p = 0.96
ns
Motabolite level
72h_C
72h_G

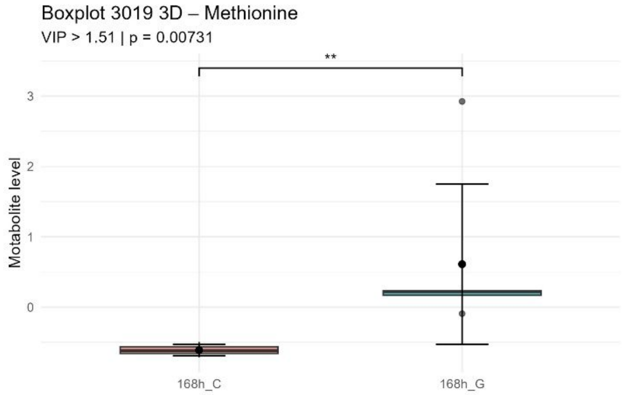
Boxplot 3019 3D – Methionine
VIP > 1.51 | p = 0.00731
**
Motabolite level
168h_C
168h_G

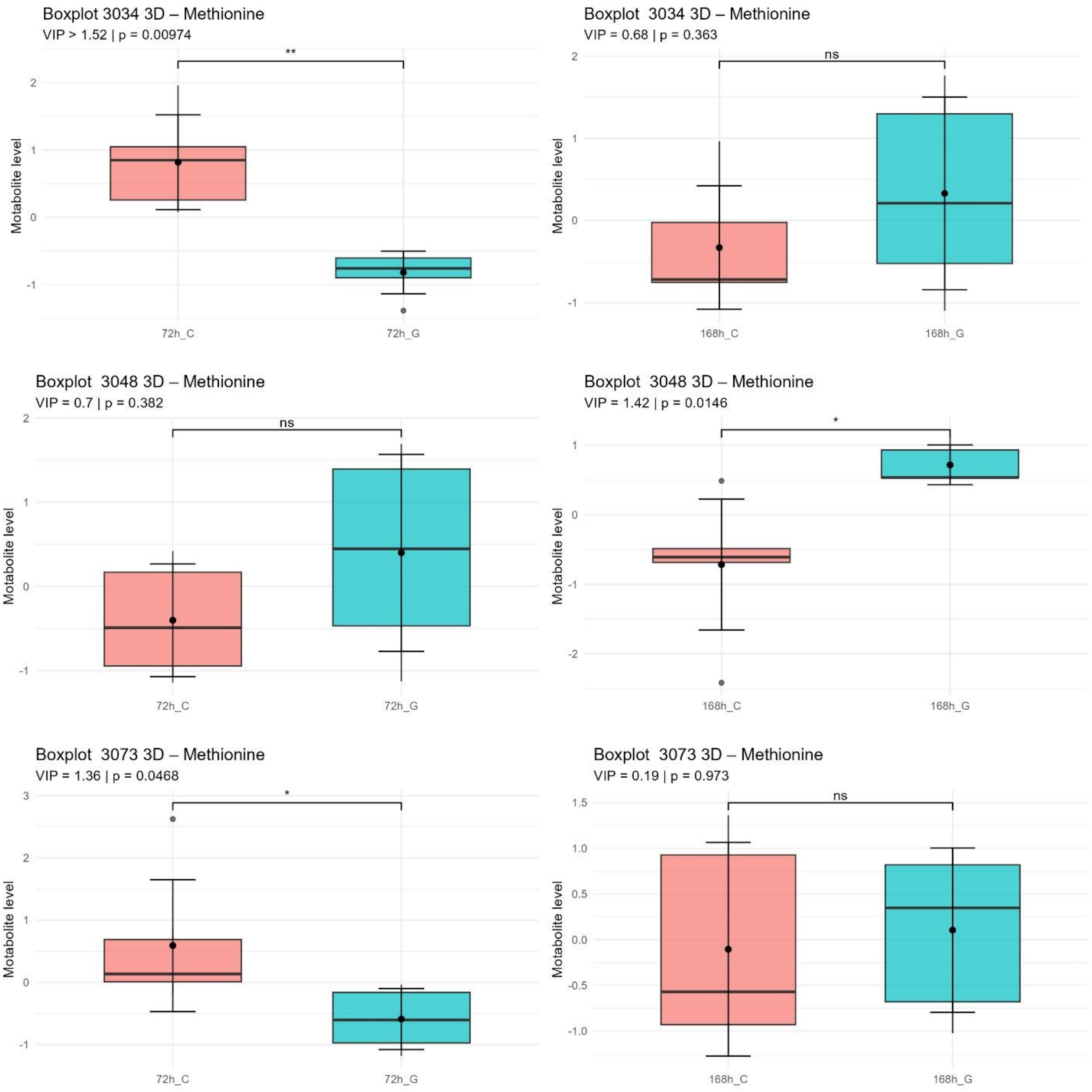

Figure S13. Change in levels of methionine between GaM treated cells and untreated control in 3D culture with VIP score and p-value (FDR). The error bars represent standard deviation (SD).

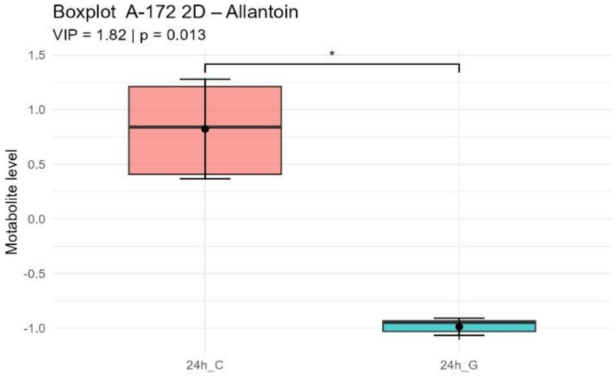
Boxplot A-172 2D – Allantoin
VIP = 1.82 | p = 0.013
*
Motabolite level
1.5
1.0
0.5
0.0
-0.5
-1.0
24h_C
24h_G

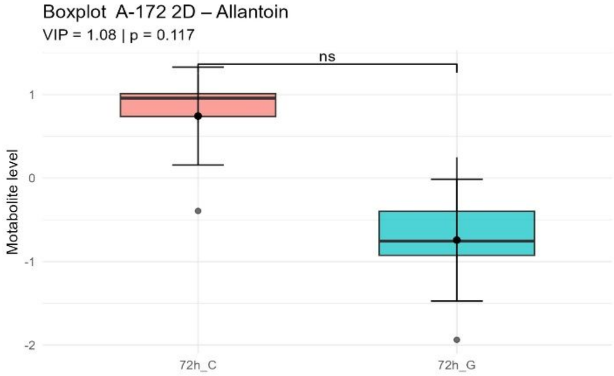
Boxplot A-172 2D – Allantoin
VIP = 1.08 | p = 0.117
ns
Motabolite level
1
0
-1
-2
72h_C
72h_G

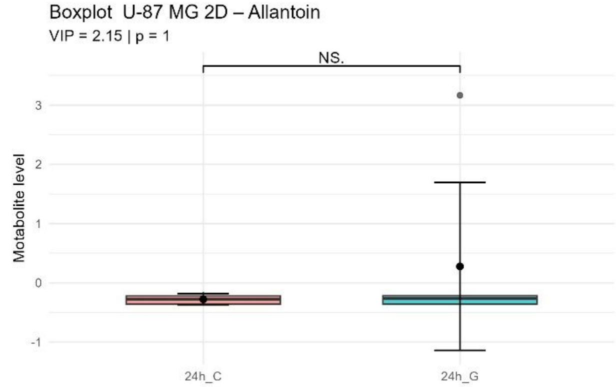
Boxplot U-87 MG 2D – Allantoin
VIP = 2.15 | p = 1
NS.
Motabolite level
3
2
1
0
-1
24h_C
24h_G

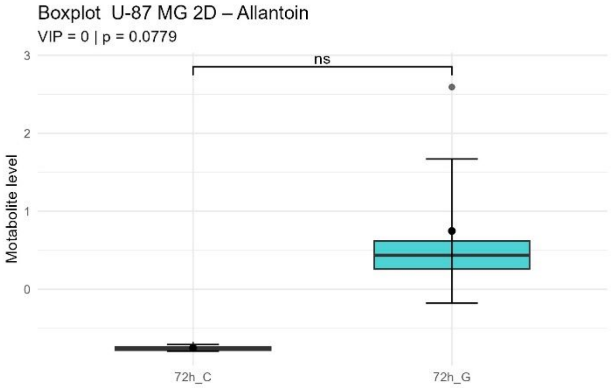
Boxplot U-87 MG 2D – Allantoin
VIP = 0 | p = 0.0779
ns
Motabolite level
3
2
1
0
72h_C
72h_G

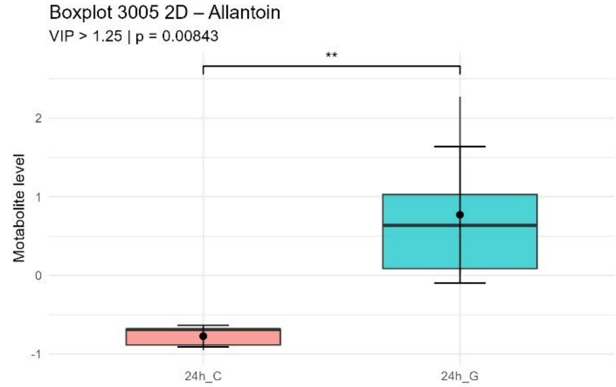
Boxplot 3005 2D – Allantoin
VIP > 1.25 | p = 0.00843
**
Motabolite level
2
1
0
-1
24h_C
24h_G

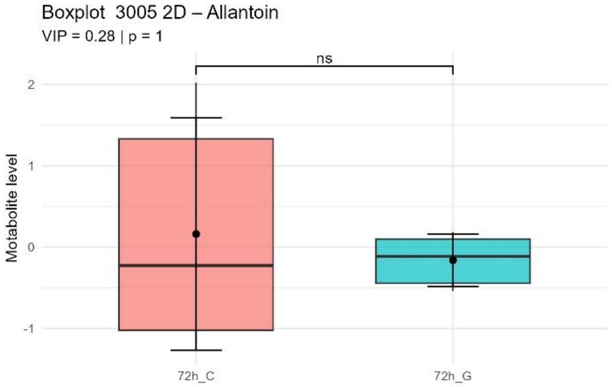
Boxplot 3005 2D – Allantoin
VIP = 0.28 | p = 1
ns
Motabolite level
2
1
0
-1
72h_C
72h_G

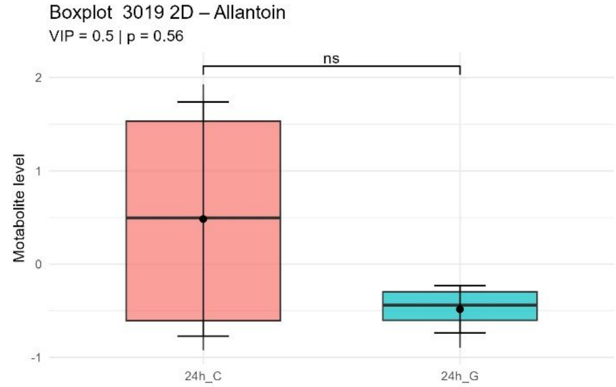
Boxplot 3019 2D – Allantoin
VIP = 0.5 | p = 0.56
ns
Motabolite level
2
1
0
-1
24h_C
24h_G

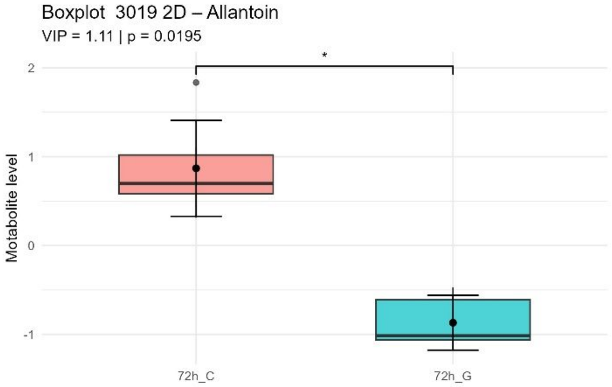
Boxplot 3019 2D – Allantoin
VIP = 1.11 | p = 0.0195
*
Motabolite level
2
1
0
-1
72h_C
72h_G

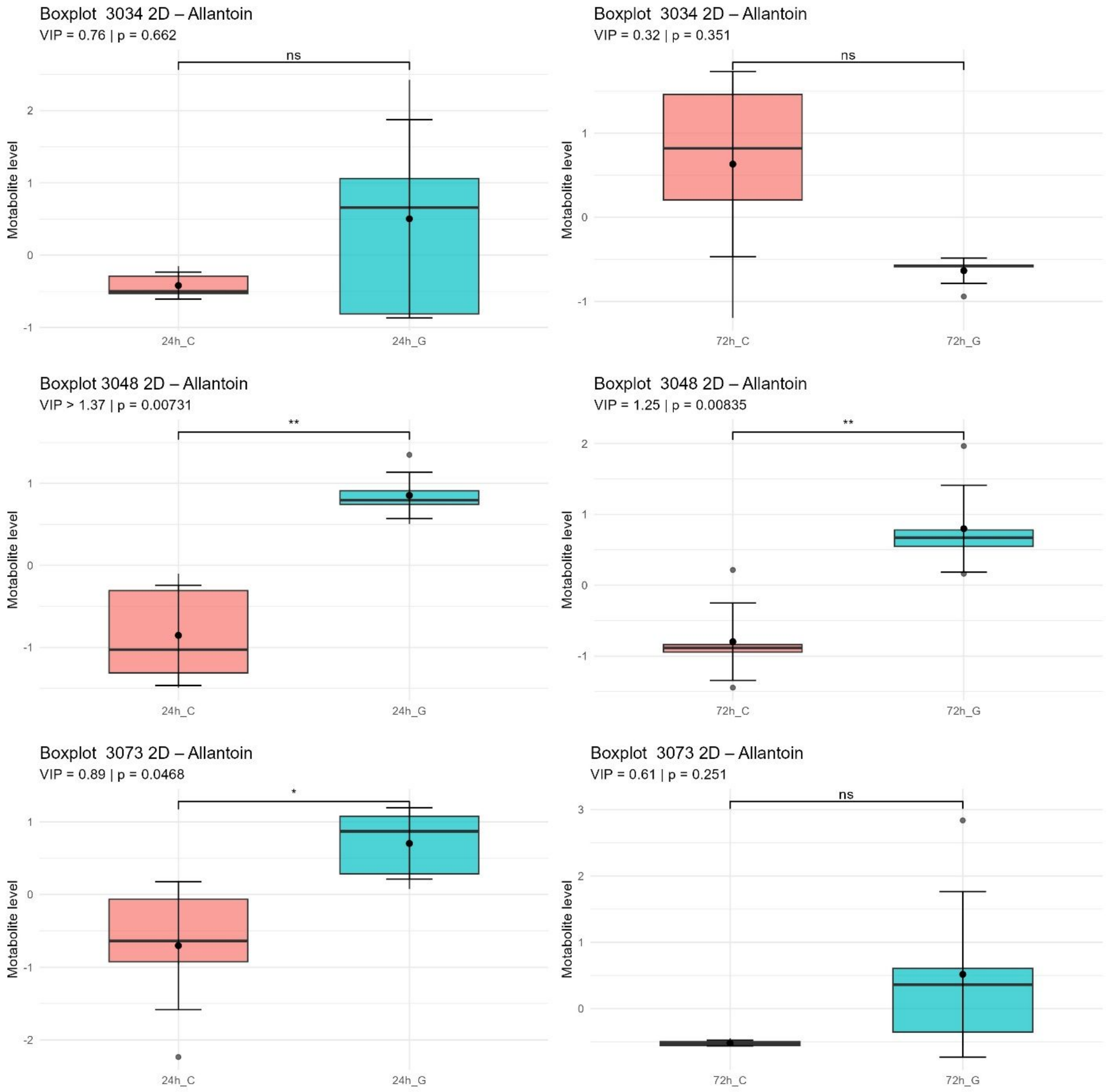

Figure S14. Change in levels of allatoin between GaM treated cells and untreated control in 2D culture with VIP score and p-value (FDR). The error bars represent standard deviation (SD).

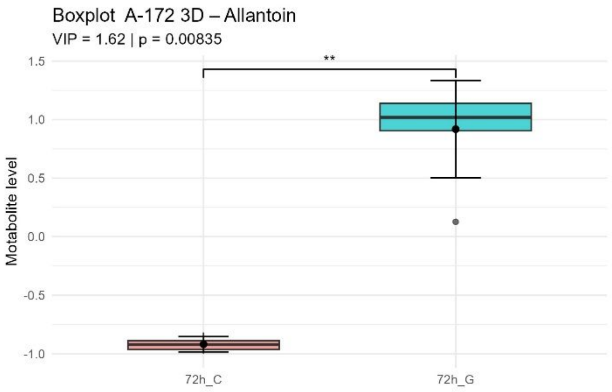
Boxplot A-172 3D – Allantoin
VIP = 1.62 | p = 0.00835
**
Motabolite level
1.5
1.0
0.5
0.0
-0.5
-1.0
72h_C
72h_G

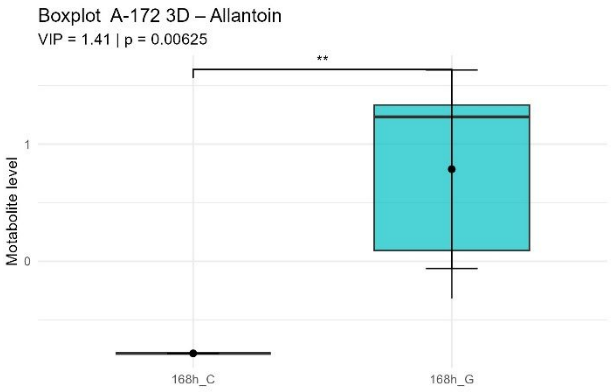
Boxplot A-172 3D – Allantoin
VIP = 1.41 | p = 0.00625
**
Motabolite level
1
0
168h_C
168h_G

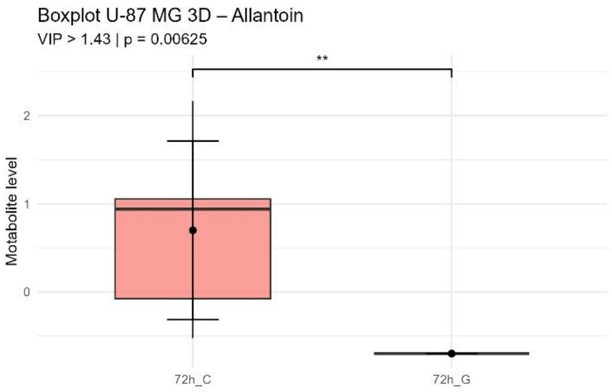
Boxplot U-87 MG 3D – Allantoin
VIP > 1.43 | p = 0.00625
**
Motabolite level
2
1
0
72h_C
72h_G

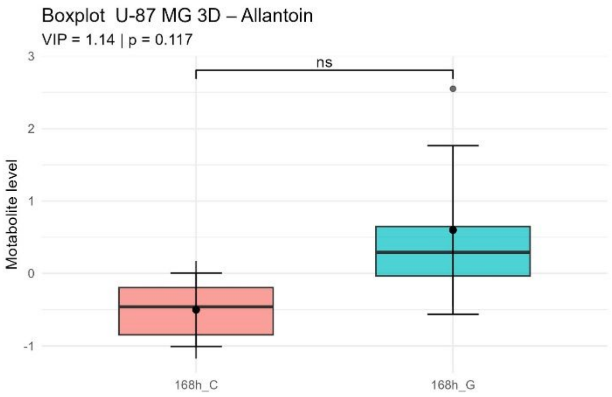
Boxplot U-87 MG 3D – Allantoin
VIP = 1.14 | p = 0.117
ns
Motabolite level
3
2
1
0
-1
168h_C
168h_G

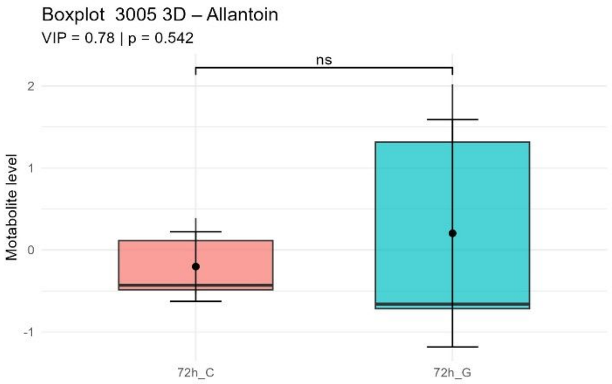
Boxplot 3005 3D – Allantoin
VIP = 0.78 | p = 0.542
ns
Motabolite level
2
1
0
-1
72h_C
72h_G

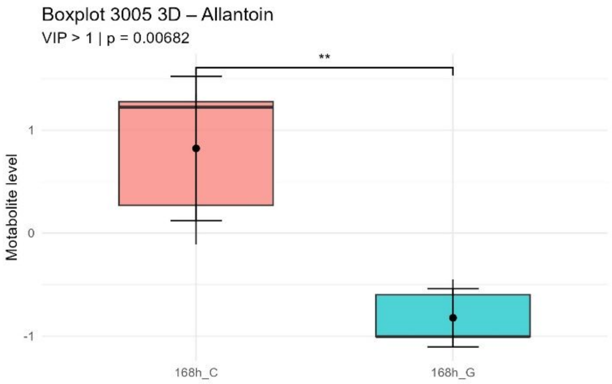
Boxplot 3005 3D – Allantoin
VIP > 1 | p = 0.00682
**
Motabolite level
1
0
-1
168h_C
168h_G

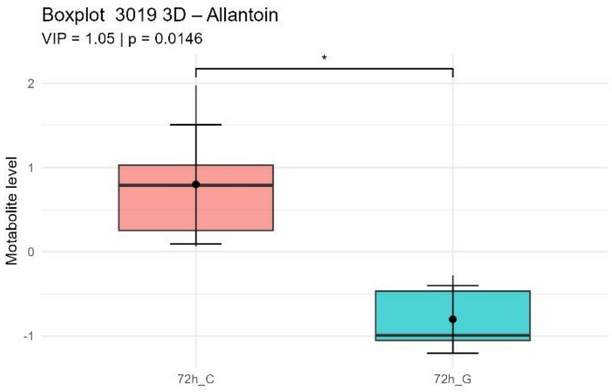
Boxplot 3019 3D – Allantoin
VIP = 1.05 | p = 0.0146
*
Motabolite level
2
1
0
-1
72h_C
72h_G

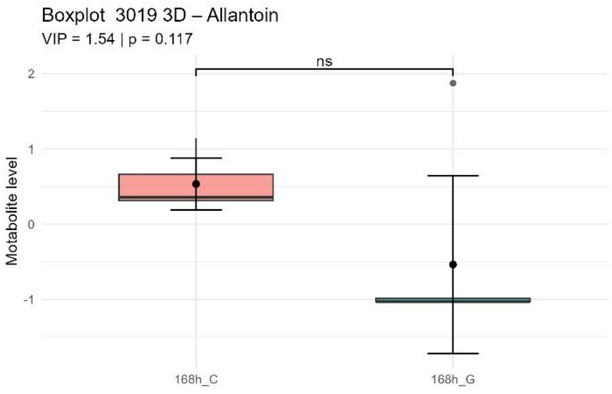
Boxplot 3019 3D – Allantoin
VIP = 1.54 | p = 0.117
ns
Motabolite level
2
1
0
-1
168h_C
168h_G

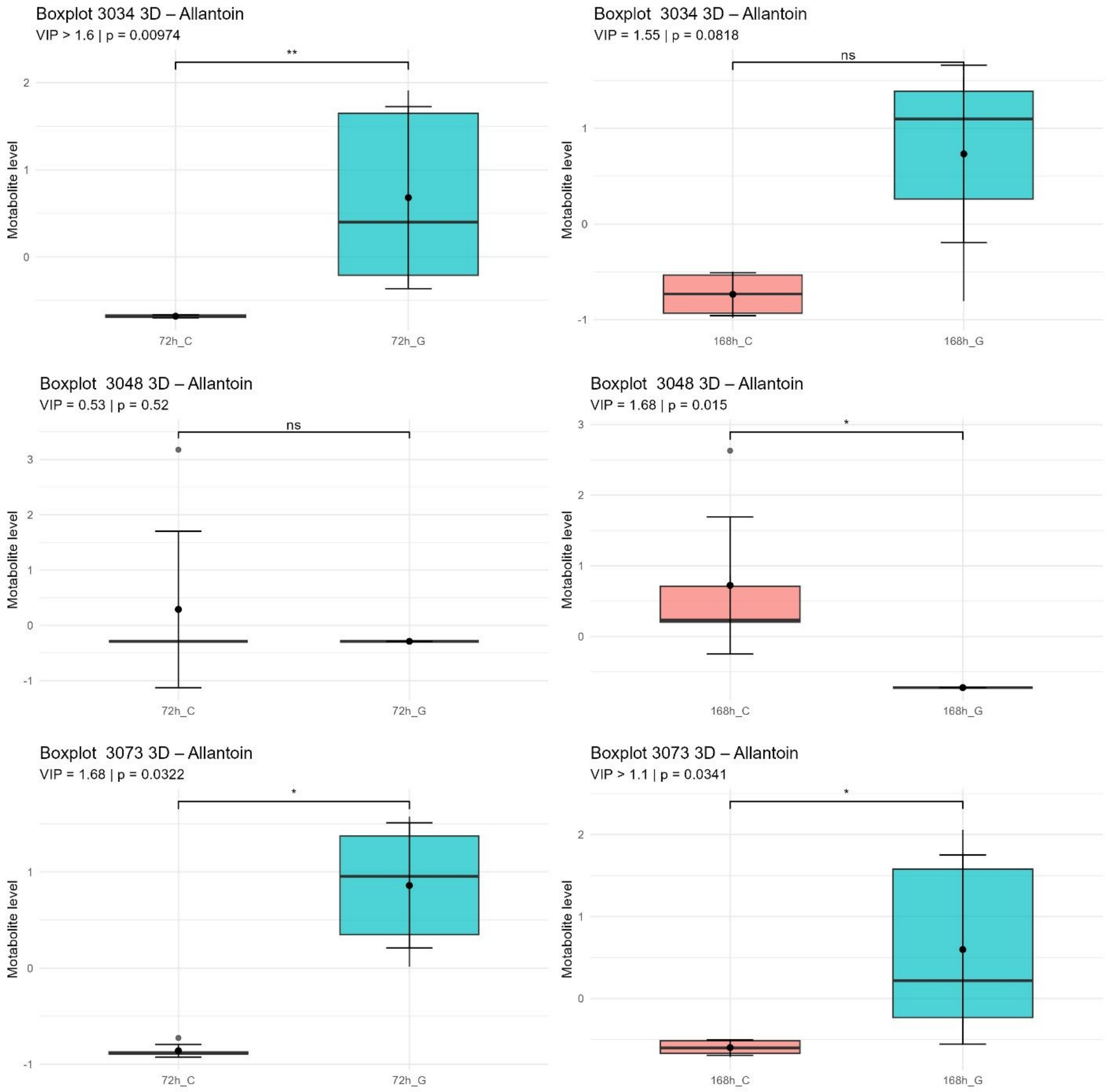

Figure S15. Change in levels of allatoin between GaM treated cells and untreated control in 3D culture with VIP score and p-value (FDR). The error bars represent standard deviation (SD).

Code for R:

Code S1. Dose-response curve with IC90, IC50 and IC10 calculations

```
library(tidyverse)

library(drc)

library(stringr)

df_long <- df_raw %>%

  pivot_longer(cols = everything(), names_to = "Concentration", values_to = "Absorbance") %>%

  mutate(Concentration = str_remove(Concentration, "^X")) %>%

  mutate(Concentration = as.numeric(Concentration))

df_clean <- df_long %>%

  group_by(Concentration) %>%

  mutate(

    mean_abs = mean(Absorbance, na.rm = TRUE),

    sd_abs = sd(Absorbance, na.rm = TRUE),

    rel_sd = (sd_abs / mean_abs) * 100

  ) %>%

  filter(rel_sd <= 15 | abs(Absorbance - mean_abs) <= 1.5 * sd_abs) %>%

  ungroup()

df_summary <- df_clean %>%

  group_by(Concentration) %>%

  summarise(

    mean_abs = mean(Absorbance, na.rm = TRUE),

    sd_abs = sd(Absorbance, na.rm = TRUE),

    .groups = "drop"

  )

control_mean <- df_summary %>% filter(Concentration == 0) %>% pull(mean_abs)
```

```
df_summary <- df_summary %>%
  mutate(
    Survival = (mean_abs / control_mean) * 100,
    Survival_sd = (sd_abs / control_mean) * 100
  )

model <- drm(Survival ~ Concentration, data = df_summary, fct = LL.4())

ic50 <- ED(model, 50, interval = "none")
ic10 <- ED(model, 10, interval = "none")
ic90 <- ED(model, 90, interval = "none")
ic_values <- ED(model, c(10, 50, 90), interval = "none")
df_ic <- data.frame(
  Parameter = c("IC10", "IC50", "IC90"),
  Value_uM = round(ic_values, 2)
)
ggplot(df_summary, aes(x = Concentration, y = Survival)) +
  geom_point(size = 3, color = "steelblue") +
  geom_errorbar(aes(ymin = Survival - Survival_sd, ymax = Survival + Survival_sd),
                width = 2, color = "gray50") +
  stat_smooth(method = "drm", formula = y ~ x,
              method.args = list(fct = LL.4()), se = FALSE, color = "red") +
  geom_vline(xintercept = ic10[1], linetype = "dotted", color = "blue") +
  geom_vline(xintercept = ic50[1], linetype = "dashed", color = "red")  +
  geom_vline(xintercept = ic90[1], linetype = "dotted", color = "purple") +
```

```
  annotate("text", x = ic10[1], y = 90, label = paste("IC10 =", round(ic10[1], 2)), color = "blue") +
  annotate("text", x = ic50[1], y = 50, label = paste("IC50 =", round(ic50[1], 2)), color = "red" )  +
  annotate("text", x = ic90[1], y = 10, label = paste("IC90 =", round(ic90[1], 2)), color = "purple")
+
  labs(
    title = "U-87MG 3D Viability (CellTiter Glo Assay)",
    x = "Concentration [µM]",
    y = "% Viability compared to control"
  ) +
  theme_minimal()
```

Code S2. T-test for TFRC level determination and significance of changes determination in 2D and 3D control

```
library(readr)
library(tidyverse)
library(ggplot2)
library(ggpubr)
library(dplyr)
library(FSA)

cell_data <- cell_data %>% rename(Group = 1)

data_long <- cell_data %>%
  pivot_longer(cols = -Group, names_to = "CellLine", values_to = "Value") %>%
  mutate(Group = factor(Group, levels = c("C_2D", "C_3D")))

ttest_results <- data_long %>%
  group_by(CellLine) %>%
  summarise(
    p_value = t.test(Value ~ Group)$p.value
  ) %>%
  mutate(
    stars = case_when(
      p_value <= 0.001 ~ "***",
      p_value <= 0.01  ~ "**",
      p_value <= 0.05  ~ "*",
```

```
    )
  )

pval_data <- ttest_results %>%
  mutate(
    group1 = "2D",
    group2 = "3D",
    y.position = 1.1 * max(data_long$Value, na.rm = TRUE)
  ) %>%
  select(CellLine, group1, group2, y.position, stars)

ggplot(data_long, aes(x = Group, y = Value, fill = Group)) +
  stat_summary(fun = mean, geom = "bar", width = 0.7, alpha = 0.8) +
  stat_summary(fun.data = mean_sdl, geom = "errorbar", width = 0.2) +
  stat_pvalue_manual(
    pval_data,
    label = "stars",
    xmin = "group1",
    xmax = "group2",
    y.position = "y.position"
  ) +
  facet_wrap(~CellLine, scales = "free_y") +
  theme_minimal() +
  labs(title = "TFRC in non-treated cells" ,
       subtitle = "Test t-Studenta + p-value",
       x = NULL, y = "TFRC/ Total Protein") +
```

```
theme(
  axis.text.x = element_blank(),
  axis.ticks.x = element_blank(),
  legend.position = c(0.95, 0.05),
  legend.justification = c(1, 0),
  plot.title = element_text(hjust = 0.5)
)
```

Code S3. Pearson correlation test between IC10 and TFRC level in 2D and 3D cell lines.

```
library(tidyverse)
library(ggpubr)

tfrc <- df[1:3, -1] %>%
  pivot_longer(cols = everything(), names_to = "CellLine", values_to = "TFRC") %>%
  mutate(gene = "TFRC")

ic10 <- df[4:6, -1] %>%
  pivot_longer(cols = everything(), names_to = "CellLine", values_to = "IC10") %>%
  mutate(gene = "IC10")

tfrc_avg <- tfrc %>%
  group_by(CellLine) %>%
  summarise(TFRC = mean(TFRC, na.rm = TRUE))

ic10_avg <- ic10 %>%
  group_by(CellLine) %>%
  summarise(IC10 = mean(IC10, na.rm = TRUE))

tfrc_avg <- tfrc %>% group_by(CellLine) %>% summarise(TFRC = mean(TFRC, na.rm = TRUE))
ic50_avg <- ic10 %>% group_by(CellLine) %>% summarise(IC50 = mean(IC10, na.rm = TRUE))

df_corr <- left_join(tfrc_avg, ic10_avg, by = "CellLine") %>% drop_na()
cor.test(df_corr$TFRC, df_corr$IC10, method = "pearson")
```

```
ggplot(df_corr, aes(x = TFRC, y = IC10)) +
  geom_point(size = 3.2, color = "steelblue") +
  geom_smooth(method = "lm", se = FALSE, color = "darkred", linewidth = 1) +
  # jeśli chcesz klasyczny geom_text (większa czcionka):
  # geom_text(aes(label = CellLine), vjust = -0.9, size = 4.2, fontface = "italic")
  # albo lepiej – etykiety, które się nie nakładają:
  ggrepel::geom_text_repel(aes(label = CellLine), size = 4.2, fontface = "italic",
                           max.overlaps = Inf, box.padding = 0.25) +
  stat_cor(method = "pearson",
           label.x = min(df_corr$TFRC, na.rm = TRUE),
           label.y = max(df_corr$IC10,  na.rm = TRUE),
           size = 5) +  # << rozmiar napisu R i p
  labs(
    title = "3D Correlation between TFRC expression and IC10",
    x = "TFRC level (mean)",
    y = "IC10 (mean)"
  ) +
  theme_minimal(base_size = 16) +   # << bazowy rozmiar czcionki
  theme(
    plot.title  = element_text(size = 18, face = "bold"),
    axis.title  = element_text(size = 15),
    axis.text   = element_text(size = 13)
  )
```

Code S.4 TFRC level determination in treated cells and untreated control, Kurskal-Wallis test with dunn post-hoc.

```
library(readr)
library(tidyverse)
library(ggplot2)
library(ggpubr)
library(dplyr)
library(FSA)
data_long_3D <- tfrc1_3D %>%
  pivot_longer(cols = -Group, names_to = "CellLine", values_to = "TfRC_per_protein") %>%
  mutate(Group = factor(Group, levels = c("C", "72h_G", "168h_G")))

dunn_all_3D <- data_long_3D %>%
  group_by(CellLine) %>%
  group_map(~{
    kw <- kruskal.test(TfRC_per_protein ~ Group, data = .x)

    if (kw$p.value > 0.05) return(NULL)

    dunn <- dunnTest(TfRC_per_protein ~ Group, data = .x, method = "bh")$res

    dunn %>%
      separate(Comparison, into = c("group1", "group2"), sep = " - ") %>%
      mutate(
        CellLine = .y$CellLine,
        y.position = max(.x$TfRC_per_protein, na.rm = TRUE) * 1.1 + row_number() * 0.05,
        stars = case_when(
          P.adj <= 0.001 ~ "***",
```

```
      P.adj <= 0.01  ~ "**",
      P.adj <= 0.05  ~ "*",
      TRUE         ~ NA_character_
     )
    ) %>%
    drop_na(stars) %>%
    select(group1, group2, y.position, stars, CellLine)
  }) %>%
  bind_rows()

ggplot(data_long_3D, aes(x = Group, y = TfRC_per_protein, fill = Group)) +
  stat_summary(fun = mean, geom = "bar", width = 0.7, alpha = 0.8) +
  stat_summary(fun.data = mean_sdl, geom = "errorbar", width = 0.2) +
  stat_pvalue_manual(
   dunn_all_3D,
   label = "stars",
   xmin = "group1",
   xmax = "group2",
   y.position = "y.position",
   tip.length = 0.01
  ) +
  facet_wrap(~CellLine, scales = "free_y") +
  theme_minimal() +
  labs(title = "TFRC of 3D cells after treatement",
     subtitle = "Kruskal-Wallis + Dunn post-hoc (p-value)",
     x = "", y = "TFRC / Total Protein") +
```

```
theme(
  plot.title = element_text(hjust = 0.5),
  axis.text.x = element_blank(),
  axis.ticks.x = element_blank(),
  legend.position = c(0.95, 0.05),
  legend.justification = c(1, 0)
)
```

Code S5. The Oxygen Consuption Rate curve in time.

```
library(tidyverse)
names(df)[1] <- "ID"

df <- df %>%
  separate(ID, into = c("CellLine", "Condition"), sep = "_", extra = "merge")

names(df) <- gsub("^X", "", names(df))

df_long <- df %>%
  pivot_longer(
    cols = -c(CellLine, Condition),
    names_to = "Time",
    values_to = "Absorbance"
  )

df_long$Time <- as.numeric(df_long$Time)

df_summary <- df_long %>%
  group_by(CellLine, Condition, Time) %>%
  summarise(
    mean_abs = mean(Absorbance, na.rm = TRUE),
    sd_abs = sd(Absorbance, na.rm = TRUE),
    .groups = "drop"
  )
```

```
cell_to_plot <- "A-172"

df_plot <- df_summary %>% filter(CellLine == cell_to_plot)

ggplot(df_plot, aes(x = Time, y = mean_abs, color = Condition)) +
  geom_line(size = 1.2) +   # grubsza linia
  geom_point(size = 3) +    # większe kropki
  geom_errorbar(aes(ymin = mean_abs - sd_abs, ymax = mean_abs + sd_abs),
                width = 1, size = 0.6) +  # cieńsze słupki błędu
  scale_x_continuous(breaks = seq(0, 135, by = 5)) +
  labs(
    title = paste("Oxygen Consumption Rate -", cell_to_plot),
    x = "Time (min)",
    y = "Absorbancce (mean ± SD)"
  ) +
  theme_minimal()
```

Code S6 Wilcox test with FDR correction and selection of metabolites with VIP > 1 and adj. p-value (FDR).

```
names(vip)[1] <- "Metabolite"

metabolite_data <- df %>%
  select(-NAME, -Group)

log_data <- log10(metabolite_data + 1)  # dodajemy 1, by uniknąć log(0)

scaled_data <- scale(log_data)

df_scaled <- bind_cols(df %>% select(NAME, Group), as.data.frame(scaled_data))

df_long <- df_scaled %>%
  pivot_longer(-c(NAME, Group), names_to = "Metabolite", values_to = "Value")

stats <- df_long %>%
  group_by(Metabolite) %>%
  summarise(p_value = wilcox.test(Value ~ Group)$p.value) %>%
  mutate(p_adj = p.adjust(p_value, method = "fdr"))

vip_df <- vip %>%
  select(Metabolite, VIP = `Comp. 1`)

final <- stats %>%
```

```
  inner_join(vip_df, by = "Metabolite") %>%
  filter(p_adj < 0.05 & VIP > 1) %>%
  mutate(
    stars = case_when(
      p_adj <= 0.001 ~ "***",
      p_adj <= 0.01  ~ "**",
      p_adj <= 0.05  ~ "*"
    )
  )

selected_metabolites <- final$Metabolite

all_meta <- stats %>%
  inner_join(vip_df, by = "Metabolite")

for (met in selected_metabolites) {
  df_met <- df_long %>% filter(Metabolite == met)

  if (all(c("72h_C", "72h_G") %in% unique(df_met$Group))) {

    y_max <- max(df_met$Value, na.rm = TRUE)
    p_val <- all_meta$p_adj[all_meta$Metabolite == met]

    p <- ggplot(df_met, aes(x = Group, y = Value, fill = Group)) +
      geom_boxplot(width = 0.6, alpha = 0.7) +
      stat_summary(fun = mean, geom = "point", shape = 20, size = 3, color = "black") +
```

```
    stat_summary(fun.data = mean_sdl, fun.args = list(mult = 1),
                 geom = "errorbar", width = 0.2, color = "black") +
    geom_signif(
      annotations = ifelse(p_val <= 0.001, "***",
                           ifelse(p_val <= 0.01, "**",
                                  ifelse(p_val <= 0.05, "*", "ns"))),
      y_position = y_max * 1.1,
      xmin = 1, xmax = 2
    ) +
    labs(title = paste("Boxplot  3034 3D –", met),
         subtitle = paste("VIP =", round(all_meta$VIP[all_meta$Metabolite == met], 2),
                          "| p =", signif(all_meta$p_adj[all_meta$Metabolite == met], 3)),
         y = "Motabolite level", x = "") +
    theme_minimal() +
    theme(legend.position = "none")

  ggsave(filename = paste0("boxplot_", met, ".png"), plot = p, width = 6, height = 4, dpi = 300)
 }
}
```
